# *In-situ* forming ultra-mechanically sensitive materials for high-sensitivity stretchable fiber strain sensors

**DOI:** 10.1093/nsr/nwae158

**Published:** 2024-04-30

**Authors:** Rouhui Yu, Changxian Wang, Xiangheng Du, Xiaowen Bai, Yongzhong Tong, Huifang Chen, Xuemei Sun, Jing Yang, Naoji Matsuhisa, Huisheng Peng, Meifang Zhu, Shaowu Pan

**Affiliations:** State Key Laboratory for Modification of Chemical Fibers and Polymer Materials, College of Materials Science and Engineering, Donghua University, Shanghai 201620, China; MOE Key Lab of Disaster Forecast and Control in Engineering, School of Mechanics and Construction Engineering, Jinan University, Guangzhou 510632, China; State Key Laboratory for Modification of Chemical Fibers and Polymer Materials, College of Materials Science and Engineering, Donghua University, Shanghai 201620, China; State Key Laboratory for Modification of Chemical Fibers and Polymer Materials, College of Materials Science and Engineering, Donghua University, Shanghai 201620, China; State Key Laboratory for Modification of Chemical Fibers and Polymer Materials, College of Materials Science and Engineering, Donghua University, Shanghai 201620, China; State Key Laboratory for Modification of Chemical Fibers and Polymer Materials, College of Materials Science and Engineering, Donghua University, Shanghai 201620, China; State Key Laboratory of Molecular Engineering of Polymers, Department of Macromolecular Science, and Institute of Fiber Materials and Devices, Fudan University, Shanghai 200438, China; Department of Cardiology, Shanghai Xuhui Central Hospital, Zhongshan-Xuhui Hospital, Fudan University, Shanghai 200031, China; Research Center for Advanced Science and Technology, and Institute of Industrial Science, The University of Tokyo, Tokyo 153-8505, Japan; State Key Laboratory of Molecular Engineering of Polymers, Department of Macromolecular Science, and Institute of Fiber Materials and Devices, Fudan University, Shanghai 200438, China; State Key Laboratory for Modification of Chemical Fibers and Polymer Materials, College of Materials Science and Engineering, Donghua University, Shanghai 201620, China; State Key Laboratory for Modification of Chemical Fibers and Polymer Materials, College of Materials Science and Engineering, Donghua University, Shanghai 201620, China

**Keywords:** fiber, strain sensor, stretchable, wearable, healthcare

## Abstract

Fiber electronics with flexible and weavable features can be easily integrated into textiles for wearable applications. However, due to small sizes and curved surfaces of fiber materials, it remains challenging to load robust active layers, thus hindering production of high-sensitivity fiber strain sensors. Herein, functional sensing materials are firmly anchored on the fiber surface *in-situ* through a hydrolytic condensation process. The anchoring sensing layer with robust interfacial adhesion is ultra-mechanically sensitive, which significantly improves the sensitivity of strain sensors due to the easy generation of microcracks during stretching. The resulting stretchable fiber sensors simultaneously possess an ultra-low strain detection limit of 0.05%, a high stretchability of 100%, and a high gauge factor of 433.6, giving 254-folds enhancement in sensitivity. Additionally, these fiber sensors are soft and lightweight, enabling them to be attached onto skin or woven into clothes for recording physiological signals, e.g. pulse wave velocity has been effectively obtained by them. As a demonstration, a fiber sensor-based wearable smart healthcare system is designed to monitor and transmit health status for timely intervention. This work presents an effective strategy for developing high-performance fiber strain sensors as well as other stretchable electronic devices.

## Teaser

A high-sensitivity and scalable stretchable fiber strain sensor is developed through the *in situ* formation of ultra-mechanically sensitive materials, achieving a wearable healthcare system for real-time health monitoring.

## INTRODUCTION

Fiber electronics are considered to be one of the most promising devices for wearable applications due to their one-dimensional configuration, high flexibility and light weight, which allow them to be easily integrated into textiles [1–11]. Versatile functions, such as smart sensing, energy harvesting/storage, and display, are well developed in fiber electronics [12–20]. Stretchable strain sensors that can convert mechanical deformations into electrical signals are particularly attractive because of their impressive applications in smart healthcare, human–machine interfaces, soft robotics, and so on [21–24]. For the wearable smart healthcare system, the embedded fiber strain sensors detect physiological signals and human motion, such as respiration, pulse and joint movement. Subsequently, the electrical signals generated are analyzed to evaluate health status, thus enabling wearable healthcare applications [13,25,26]. Sensitivity is one of the most important parameters that determines detection accuracy and application scenarios, which is mainly related to the active materials and structures of sensors. Unlike traditional thin film-based strain sensors, fiber strain sensors show unique features of small sizes and curved surfaces, which make them challenging to effectively load active materials. For example, the active material can be deposited onto the flat film to optimize sensing performance using 3D printing and laser direct writing techniques [27–29]. However, these techniques may not be suitable for fiber materials. There is a need for the development of effective methods to load active materials onto fiber materials in order to enhance the performance of fiber strain sensors.

**Figure 1.**
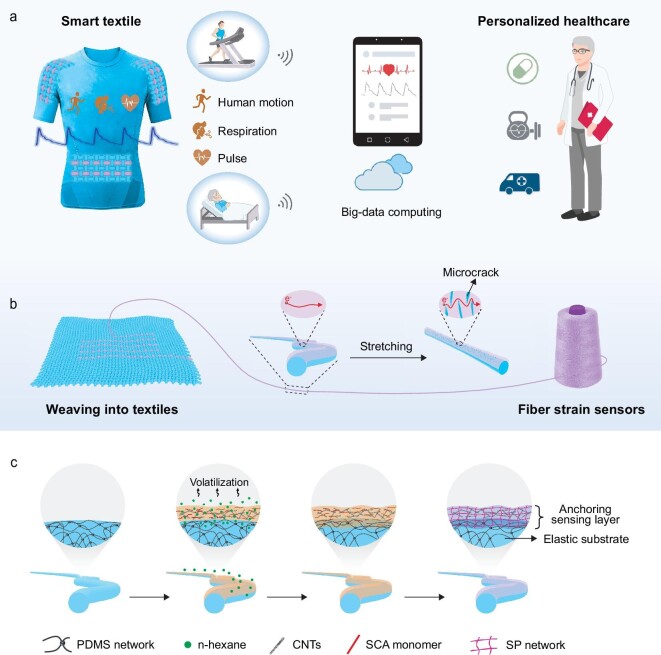
Wearable healthcare system based on stretchable fiber strain sensors, including application scenarios, sensor materials and structures. (a) Schematic of smart textiles for wearable healthcare systems based on stretchable fiber strain sensors. (b) Stretchable fiber strain sensors woven into textiles. The sensing mechanism is based on the generation of microcracks in the sensing layer upon stretching. (c) Schematic illustration of the fabrication process of fiber strain sensors based on the formation of anchoring sensing materials *in-situ*.

Generally, resistive-type stretchable strain sensors are composed of a conductive sensing material and an elastomeric substrate. Conductive sensing materials are usually non-stretchable. During stretching, the conductive sensing material undergoes structural changes, resulting in a variation in electrical resistance. Upon the release of the applied strain, the conductive sensing materials regain their structure with the aid of the elastomeric polymer, thereby restoring the electrical resistance of those sensors. Thus, the sensitivity is defined as *GF* = (∆*R*/*R*_0_)/ε, where *ΔR/R_0_* refers to the relative resistance change with applied strain *ε*. The degree of deformation in sensing materials under tensile strain largely determines the sensitivity of fiber strain sensors. Typically, highly sensitive strain sensors require materials capable of performing significant structural deformation under a tiny strain. Till now, fiber strain sensors have been fabricated by filling or coating conductive materials, including carbon-based nanomaterials, metal nanowires, metal nanofilms and conductive polymers, through techniques such as wet-spinning, dip-coating and thermal evaporation deposition [30–32]. Sensitivities of conductive filler filled fiber sensors are relatively low due to moderate relative electrical resistance change due to the tunneling effect [33]. For the surface coating–based fiber strain sensors, microcracks are generated in the conductive materials under tensile stress due to the mechanical mismatch between elastomeric substrate and rigid conductive material. The formation of these microcracks increases the device's electrical resistance, thereby acquiring sensing functionality. However, the entanglement of conductive materials impedes the formation of microcracks, particularly in tiny tensile strains, which result in low sensitivity [34–37]. In addition, physically surface–coated conductive materials are prone to fall off during the weaving and integration process of fiber sensors. On the other hand, developing mechanically engineered materials have been proven to be an effective method for improving sensor sensitivity [38]. The mechanically engineered substrates, including auxetic mechanical metamaterials, mechanically heterogeneous films and structured microfibers, delicately control the structural deformation of sensing materials, typically resulting in longer and wider microcracks [32,39,40]. However, it is still a challenge to develop mechanically engineered thin fibers to enhance sensitivity. Therefore, constructing mechanically engineered sensing materials that promote microcrack generation and establish a robust interface with the substrate is key to developing high-performance fiber strain sensors, which is of great significance for wearable healthcare systems (Fig. 1a).

Herein, we report a high-sensitivity stretchable fiber strain sensor based on the formation of anchoring sensing materials *in-situ*. Due to the diffusion of silane coupling agent monomer into the fiber matrix, the ultra-mechanically sensitive sensing layer is firmly anchored in the elastic fiber through a hydrolytic condensation reaction, making the sensor easier to generate microcracks for high sensitivity (Fig. 1b and c). The resulting stretchable fiber strain sensors simultaneously possess an ultra-low strain detection limit of 0.05%, a high stretchability of 100%, and a high gauge factor of 433.6, which can monitor tiny strains, e.g. vibration, pulse and respiration. The highly-sensitive yet robust fiber strain sensors can be used to measure pulse wave velocity (PWV) for assessing cardiovascular health. In addition, the flexible, miniature, and lightweight fiber strain sensors can be well integrated into clothing to develop wearable healthcare systems for the diagnosis of respiratory disorders, reporting health status for real-time treatment.

## RESULTS AND DISCUSSION

**Figure 2.**
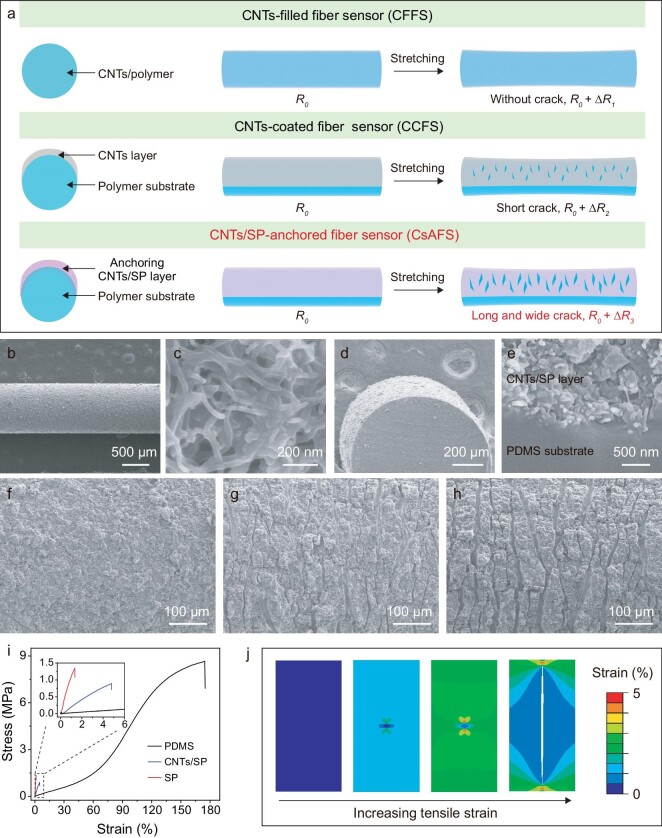
Structures and mechanism of stretchable fiber strain sensors. (a) Schematic illustration of three kinds of fiber strain sensors, including CNTs-filled fiber sensor (CFFS), CNTs-coated fiber sensor (CCFS), and our proposed CNTs/SP-anchored fiber sensor (CsAFS). (b and c) SEM images of CsAFS and the enlarged view of its surface. (d and e) Cross-section SEM images of CsAFS and enlarged view of the interface between PDMS substrate and CNTs/SP sensing layer. (f–h) SEM images of CsAFS under tensile strains of 0%, 30% and 50%, respectively; the uniaxial strain was applied in a horizontal direction. (i) Mechanical properties of PDMS, SP and CNTs/SP sensing materials. (j) Simulation of the crack formation process of ultra-mechanically sensitive CNTs/SP sensing materials using finite element method; the uniaxial strain was applied in a horizontal direction.

Resistive-type stretchable fiber strain sensors are composed of an elastomeric substrate and conductive material. The essential idea of our high-sensitivity fiber strain sensors is illustrated in Fig. 2a. PDMS has been used as a substrate for stretchable strain sensors and CNTs and was chosen as the conductive material. The two most frequently-used methods for constructing fiber strain sensors, dispersing CNTs as fillers into the fiber matrix or coating CNTs onto the fiber surface, was used as a control. The CNTs-filled fiber sensors (CFFS) usually showed low sensitivity due to slight resistance change in the homogeneous conductive network. For the CNTs-coated fiber sensors (CCFSs), the entanglement of one-dimensional CNTs greatly hindered the formation of microcracks, showing a relatively low sensitivity at small strain ranges. Differing from these two traditional structures, we introduced a silane coupling agent (SCA) as the interfacial material into the sensing materials to fabricate CNTs/SP-anchored fiber sensors (CsAFS). The hydrolysis and condensation of the silane coupling agent produce siloxane polymers (SPs), anchoring an ultra-mechanically sensitive sensing layer onto the fiber surface, which induce long and wide microcracks upon stretching which leads to improved sensitivity (Fig. 2a).

In a typical experiment for the fabrication of CsAFS, 3-aminopropyltriethoxysilane, one type of silane coupling agent, was used as an interfacial bonding agent. A mixture of CNTs and 3-aminopropyltriethoxysilane in hexane solvent was deposited onto the PDMS fiber surface *via* spraying (Fig. S1). The volatile property of the hexane solvent ensures that CNTs and silane coupling agent can quickly adhere to the fiber surface after the spraying process. After that, the ethoxysilane group in 3-aminopropyltriethoxysilane hydrolyzed to form silanols, which underwent self-condensation to create siloxane polymers (Fig. S2). A Fourier transform infrared spectrometer was used to analyze the hydrolysis and self-condensation processes. The absorption band of Si-O-CH_3_ in silane at 1080 cm^−1^ disappeared, and a new absorption band appeared at 1016 cm^−1^, corresponding to the vibration mode of Si-O-Si, confirming the hydrolysis and self-condensation of 3-aminopropyltriethoxysilane to siloxane polymers (Fig. S3) [41]. In order to enhance the interface bonding between the PDMS substrate and functional sensing layer, the PDMS fiber can be first treated with oxygen plasma to introduce oxygen-containing functional groups, which can partially react with 3-aminopropyltriethoxysilane.

The surface morphology of fiber strain sensors was investigated using scanning electron microscopy. As shown in Fig. 2b and c, the conductive CNTs were entangled with each other and mostly buried in siloxane polymer to form stable conductive composites that serve as the sensing layer. The sensing layer was distributed on the fiber surface from elemental mapping images (Fig. S4). Moreover, anchoring CNTs/SP sensing layer and fiber matrix seamlessly merged together from the cross-sectional image (Fig. 2d and e). We believe that 3-aminopropyltriethoxysilane in our approach can enter the PDMS network, which brings strong adhesion between the CNTs/SP sensing layer and fiber matrix. In order to confirm this hypothesis, PDMS fibers were soaked in Nile Red/n-hexane solution and Nile Red/ethanol solution, respectively. The fluorescence microscope images showed that Nile Red molecules in the hexane can diffuse into the PDMS matrix, with fluorescence intensity increasing with the length of soaking time (Fig. S5). No fluorescence was found in the ethanol-treated sample. In other words, the hexane can swell the PDMS matrix and help the Nile Red molecules diffuse into the PDMS matrix. Based on this fact, PDMS fibers were soaked in 3-aminopropyltriethoxysilane hexane solution and then taken out for volatilization of hexane. Next, the fibers were cleaned with ethanol to remove the surface-coated 3-aminopropyltriethoxysilane. After completing the hydrolysis and condensation of 3-aminopropyltriethoxysilane, we studied the mechanical property of treated fibers. The elongation at break of treated PDMS fibers decreased and the elastic modulus increased significantly, indicating that 3-aminopropyltriethoxysilane diffused into the swollen PDMS network (Fig. S6).

We qualitatively evaluated the adhesion strength between the PDMS fiber and anchoring sensing layer. Adhesive polyimide tapes were attached to the sensing layer and then peeled off. In the CNTs/SP-anchored sensing layer-based sample, the sensing layer was firmly adhered to the fiber substrate without peeling, showing a high adhesion strength between the active materials and PDMS fiber. In contrast, CNTs in all of the control samples were almost removed by the tape (Fig. S7 and Movie S1). To further study the interaction between the anchoring sensing layer and PDMS substrate, thin film samples were fabricated using the same method as in the fiber samples, and tensile testing was carried out by using a rigid rod and epoxy resin as glue. The CNTs/SP anchoring layer was removed along with partial PDMS substrate with a peel strength of 751.8 kPa. In contrast, for the sample without SP, the CNTs layer was easily removed from the substrate with a peel strength of 18.4 kPa. This further proved that the CNTs/SP anchoring sensing layer was firmly bound with PDMS substrate (Figs S8 and S9, and Movie S2). This strong adhesion between the sensing layer and substrate is crucial for their application in wearable electronics.

In addition, the surface morphology of fiber strain sensors under mechanical deformation were recorded *via* SEM. As shown in Fig. 2f–h, microcracks were observed in CsAFS after stretching, showing longer and wider cracks compared to those in the CCFS control sample (Fig. S10). The long microcracks could help to promote variations in electrical resistance for high sensitivity applications [39]. To understand the mechanism of crack formation, we first investigated the mechanical property of the sensing layer in CsAFS by recording its strain-stress curves. The failure strains of SP and CNTs/SP composites were 1.3% and 4.8%, respectively (Fig. 2i). In comparison, PDMS substrate showed a high stretchability with an elongation at break of around 180%. The huge mechanical differences between the CNTs/SP sensing layer and elastomeric substrate induced the formation of cracks in the CNTs/SP sensing layer upon stretching. We also used the finite element method to simulate crack behavior of the sensing layer. The deformation diagram showed that crack length increased when subjected to a slight strain (Fig. 2j), potentially resulting in a notable rise in electrical resistance. The substantial change in resistance is helpful to improve the sensitivity of stretchable strain sensors.

The thickness of the CNTs/SP sensing layer in fiber strain sensors was tuned by the number of spraying times (Fig. S11), and the thickness was calculated as follows:


(1) \begin{eqnarray*} y = {\mathrm{4}}{\mathrm{.71}}x \end{eqnarray*}

where *y* is the thickness of the sensing layer and *x* is the spraying time. A series of fiber strain sensors with different thicknesses of sensing layer were prepared, and their resistance changes under tensile strain were investigated. The stretchability of fiber strain sensors decreased as the thickness of the sensing layer increased, the maximal stretchability of the fiber stain sensor was 100% (Fig. S12). After balancing stretch range and sensitivity, fiber strain sensors with a sensing layer thickness of ∼10 μm were selected for further study. The ratio of CNTs to silane coupling agent also influenced the stretchability and sensitivity of fiber strain sensors, and sensors with a ratio of CNTs to silane coupling agent of 1 : 4 showed the best overall performance considering stretchability and sensitivity (Fig. S13).

**Figure 3.**
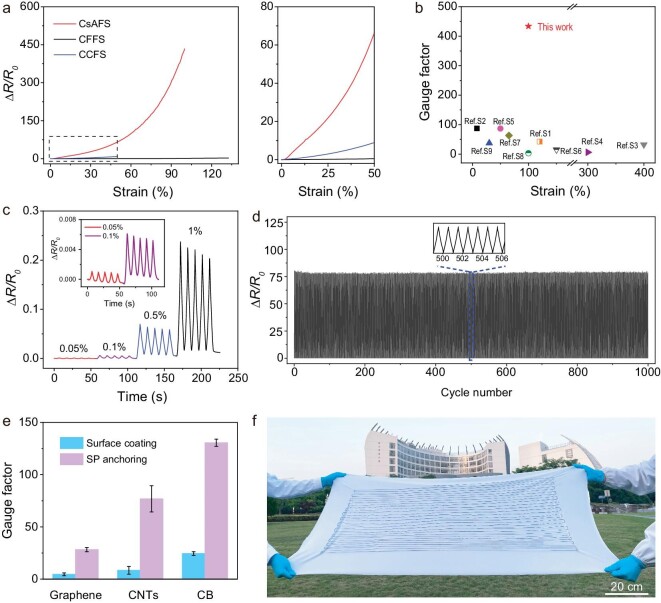
Performance of stretchable fiber strain sensors. (a) The relative resistance change curves with tensile strain for three kinds of fiber sensors (CFFS, CCFS and CsAFS). (b) Comparison of sensitivity between our fiber strain sensors and those reported in the literature. (c) Relative resistance change curves under tiny tensile strains. (d) Cyclic durability of CsAFS under tensile strain of 50%. (e) Sensitivity comparison of fiber sensors using different conductive materials based on surface coating and SP anchoring methods; the substrate was PDMS fiber, and tensile strain was 30%. (f) Photograph of an ultralong CsAFS integrated into commercial fabrics.

As shown in Fig. 3a, CsAFS showed a significant electrical resistance change compared to CFFS and CCFS as the strain increased, and CsAFS had an average gauge factor of 433.6 at 100% strain. The strain-resistance change curve can be divided into three linear regions, corresponding to three GFs: 125.5 within 50% strain, 456.3 within 50%–80% strain, and 1112.8 within 80%–100% strain (Fig. S14). In comparison, for the CFFS, CNTs is filled in the fiber matrix, and the gauge factor was only 1.7 within 100%. Therefore, the sensitivity of our proposed fiber strain sensor has been increased by 254 folds. The CCFS also showed a smaller resistance change, giving a gauge factor of 16.7 at 50% strain. The effective strain range of sensors for practical applications in wearable electronics is typically around 55% [22,42]. Therefore, our fiber strain sensor is adequate for use. Additionally, we summarized the gauge factor and strechability of other stretchable fiber strain sensors reported in the literature. These results indicate that our fiber strain sensor shows outstanding sensitivity (Fig. 3b and Table S1).

Our fiber strain sensors not only showed high stretchability, but also were able to detect minute tensile strains as low as 0.05%. Specifically, the gradual increase of minute tensile strain from 0.05% to 1% was clearly detected (Fig. 3c). This ultra-low detection limit could expand the strain sensor's application range. The durability of fiber strain sensors was investigated through continuous mechanical loading and unloading tests. It showed high repeatability after 1000 cycles at 50% strain, indicating high stability of our developed CsAFS (Fig. 3d). Additionally, the sensitivity could be well maintained for over one week (Fig. S15). Based on our strategy, other conductive materials such as carbon black and graphene can also be successfully deposited onto the curved fiber surfaces for strain sensing. For example, the graphene/SP-anchored fiber sensors and carbon black/SP-anchored fiber sensors also showed sensitivity enhancement, and the gauge factor increased to 7.1 and 5.8 times, respectively (Fig. 3e and Fig. S16). Furthermore, this sensitivity enhancement strategy can be also effective for other elastic substrates. For example, the gauge factor of TPU fiber–based CsAFS increased to 3.8 times (Fig. S17). These results indicate that our strategy is a general and effective method to improve sensitivity of stretchable strain sensors. Furthermore, it is feasible to achieve large-scale production of fiber strain sensors based on the spraying method and formation of a sensing layer *in-situ*. A 30-meter-long fiber strain sensor was fabricated using our proposed method and integrated with a commercial fabric (Fig. 3f).

The high sensitivity and flexibility of fiber strain sensors allow them to detect subtle mechanical deformation. For example, a fiber strain sensor was used to detect damping vibration. The strain sensor was attached to one end of a flexible steel sheet, while the other end was fixed. The fiber strain sensor recorded the attenuation of amplitude of the steel sheet by the value of relative resistance change when the damped vibration was generated (Fig. S18). Acoustic vibrations can be also detected by attaching a fiber strain sensor onto the surface of a loudspeaker. Synchronous resistance signals were obtained based on fiber strain sensors when the loudspeaker repeatedly made the sounds of ‘Shanghai’ (Fig. S19). Another example was that throat muscle movements as a volunteer speaks could be successfully recorded. When the volunteer successively read ‘fiber’, ‘sensor’ and ‘fiber sensor’, the features of different pronunciations could be recorded by the sensor and the characteristic waveform of sound showed good reproducibility (Fig. S20). We believe that our fiber strain sensors will have promising applications in speech recording, recognition and analysis.

**Figure 4.**
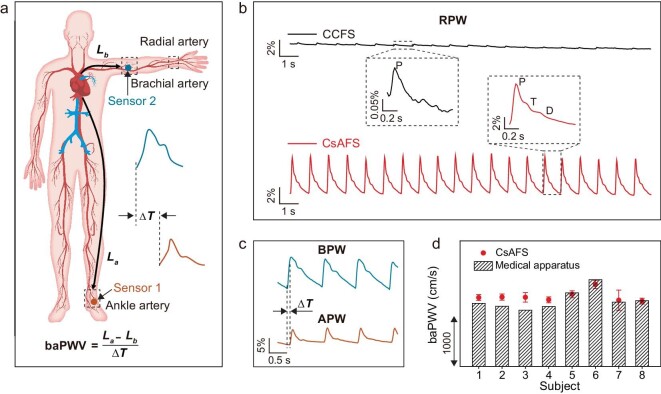
Measuring pulse wave velocity using stretchable fiber strain sensors. (a) Schematic diagram of the location of human arteries (including radial, brachial and ankle arteries), the placement of sensors relevant to baPWV, and the calculation equation for baPWV. (b) Radial pulse waveform (RPW) monitored using CCFS and CsAFS. Highly-sensitive CsAFS reliably recorded RPW, and the characteristic signals of pulse waves were all clearly observed. However, the low-sensitivity CCFS only detected P-waves, and the signal intensity was quite weak. (c) Brachial pulse waveform (BPW) and ankle pulse waveform (APW) measured by CsAFS; *ΔT* represents time interval between the wave front of BPW and that of APW. (d) The baPWV calculated by CsAFS and Omron arteriosclerosis detector in eight volunteers.

Flexible mechanical sensors can be used as on-skin sensors for non-invasive diagnosis. Pulse waves are one of the vital physiological signals and contain multiple human-health information including heart rate, blood pressure and vascular aging [43]. Pulse measurement is a medically effective way to non-invasively assess heart health. For example, brachial-ankle pulse wave velocity (baPWV), one of the most important parameters for assessing the degree of arteriosclerosis, can be obtained by wrapping the four extremities with blood pressure cuffs (Fig. S21). baPWV is calculated from following equations [44]:


(2) \begin{eqnarray*}
baPWV = \frac{L_a-L_b}{\Delta T}
\end{eqnarray*}

(3) \begin{eqnarray*}{{L}_a} = {\mathrm{ 0}}{\mathrm{.8129}}h\ {\mathrm{ + \ 12}}{\mathrm{.328}}\end{eqnarray*}

(4) \begin{eqnarray*}{{L}_b} = {\mathrm{ 0}}{\mathrm{.2195}}h{\mathrm{\ - \ 2}}{\mathrm{.0734}}\end{eqnarray*}

where *L_a_* is the path length from the heart to the ankle, *L_b_* is the path length from the heart to the brachium, *h* is the subject's height (in centimeters), and *∆T* is the time interval between the wave front of the brachial pulse waveform (BPW) and that of the ankle pulse waveform (APW) (Fig. 4a). Bulky medical apparatus and short time measurement in hospital may not be sufficient for continuous and accurate monitoring of artery pulse. Thus, utilizing flexible and miniaturized devices for non-invasive and continuous monitoring of pulse waves is another feasible alternative.

Due to the light weight, softness, small size and high sensitivity of our fiber strain sensors, it is convenient for them to integrate on human skin in order to detect weak physiological signals. Here, we attached a fiber strain sensor onto the radial artery of the wrist for recording radial pulse waveform, as shown in Fig. 4b, our developed CsAFS was capable of recording radial pulse waveform stably and continuously, and the characteristic signals of pulse waves including percussion wave (P-wave), tidal wave (T-wave), and diastolic wave (D-wave) were all clearly observed. However, the low-sensitivity CCFS only detected P-waves, and the signal intensity was quite weak. Based on the high performance of CsAFS, we employed two CsAFS to simultaneously record BPW and APW for measuring baPWV (Fig. 4c and Movie S3). As a comparison, a medical Omron arteriosclerosis detector (BP-203RPE Ⅲ) was used to measure baPWV. The baPWV of eight subjects measured by CsAFS shows the same trend as the baPWV measured by the Omron arteriosclerosis detector (Fig. 4d). Furthermore, we used Bland–Altman analysis to study the agreement of the two sets of data, and the agreement range was between −158.35 and 306.53 cm/s. All the scatter points were within this interval, indicating a good level of consistency (Fig. S22). These results indicated that CsAFS can obtain reliable baPWV for arteriosclerosis diagnosis.

Our proposed fiber strain sensors possess several merits including light weight, robustness, and weavability, making them well-suited integration into textiles for multiple applications (Fig. S23). For example, the bending degree of the elbow could also be effectively distinguished by integrating a fiber strain sensor into an elbow guard, potentially useful for human–machine interactions (Fig. S24). Respiratory rate is a basic vital sign that can be used as an indicator of potential respiratory dysfunction. Since the contraction and relaxation deformation of the abdomen occur during breathing, we integrated CsAFS into clothes at the abdomen site for monitoring the wearer's breathing state. The volunteer first jogged for 20 minutes, and their respiratory status was then subsequently monitored for analysis. The breathing of the volunteer changed from rapid to shallow with time, where the respiratory rate was around 42 breaths per minute (bpm) at the initial stage, and recovered to 18 bpm at the final stage. It needed around 4 minutes to return to normal breathing after jogging for 20 minutes (Fig. S25).

**Figure 5.**
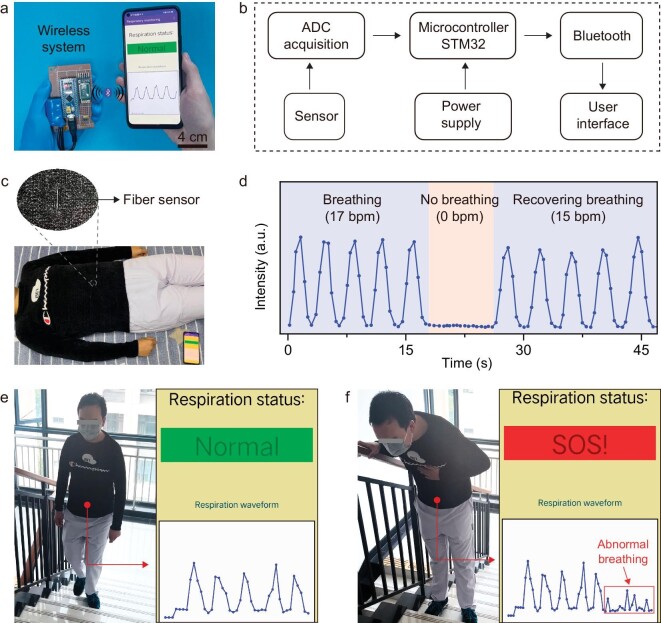
Wearable healthcare system based on fiber strain sensors for monitoring respiratory status. (a) Photograph of the wireless respiration monitoring system including power supply, microcontroller, bluetooth module, and mobile phone. (b) Circuit diagram showing the signal flow in the wireless respiration monitoring system. (c) Photograph of a volunteer wearing a sweater integrated with the fiber sensor for respiratory monitoring. (d) The electrical resistance intensity of the fiber sensor under different breathing states; the blue shaded part represents the volunteer in a normal breathing state, and the pink shaded part represents the volunteer holding breath. (e) Photograph of a volunteer under normal breathing (left), and corresponding real-time breathing signals (right). (f) Photograph of a volunteer simulating breathing during an asthma attack (left), and corresponding abnormal breathing signals displayed on a phone, prompting an alert (right).

In order to demonstrate the practical application of our stretchable fiber strain sensors, we designed a wireless respiration monitoring system. The voltage data of the fiber sensor in the circuit was continuously sampled and converted by an analog-to-digital converter, processed by microcontroller as the central processing unit, and then the analyzable data were transmitted to the phone in real time by Bluetooth (Fig. 5a and b). The circuit diagram for signal detection is shown in Fig. S26. The fiber sensor–based wearable system can conveniently and comfortably monitor the respiratory status of the volunteer in real time. The normal breathing rate of the volunteer was 15–17 bpm, and the breathing rate was 0 bpm when the volunteer deliberately held their breath to simulate apnea. Hence, the different breathing conditions could be clearly detected and recorded by our wearable sensing system. The light blue shaded area corresponded to the normal breathing of the volunteer, and the light red shaded area corresponded to the volunteer holding their breath (Fig. 5c and d, and Movie S4). Furthermore, our wearable respiratory monitoring system can be further extended to asthma detection. Breathing becomes short and rapid during asthma attacks, therefore the health condition can be assessed by measuring the frequency and amplitude of the respiratory waveform. Our wearable sensing system is suitable for different wearers by setting amplitude thresholds based on the wearer's breathing in a calm state. The system showed normal state when the volunteer was breathing normally. However, if the volunteer was experiencing an asthma attack, the amplitude of the signal decreased and fell below the threshold value, thus triggering an alarm *via* a mobile app thus enabling the sufferer to seek further medical attention (Fig. 5e and f, and Movie S5). This potential application may show great value for decentralized medical healthcare.

## CONCLUSION

In summary, we have developed a high-sensitivity fiber strain sensor based on a mechanically engineered anchoring sensing layer. The ultra-mechanically sensitive and electrically conductive sensing layers were formed *in-situ* and firmly bound to the curved fiber surfaces to generate longer microcracks after stretching. The resulting fiber strain sensor simultaneously possessed an ultra-low strain detection limit of 0.05%, large stretchability of 100%, and high sensitivity of 433.6. The flexible and robust fiber sensors were integrated with skin or clothing for tracking physiological signals. Pulse wave velocity, an important index for assessing the degree of arteriosclerosis, was reliably measured by our fiber strain sensors. Furthermore, a wearable healthcare system consisting of fiber sensors has been designed to assess human health states. This work presents an effective strategy to develop high-performance stretchable fiber sensors and other types of devices.

## Supplementary Material

nwae158_Supplemental_Files
