# Supplementary material for: In-situ forming ultra-mechanically sensitive materials for high-sensitivity stretchable fiber strain sensors
Source: Natl Sci Rev. 2024 Apr 30;11(6):nwae158. doi: 10.1093/nsr/nwae158 (PMC11177883; doi:10.1093/nsr/nwae158)
Supplement: nwae158_Supplemental_Files [file nwae158_supplemental_files.zip › Supplementary Data-r.docx]

**Supporting Information**

***In-situ* forming ultra-mechanically sensitive materials for high-sensitivity stretchable fiber strain sensors**

Rouhui Yu^1^, Changxian Wang^3^, Xiangheng Du^1^, Xiaowen Bai^1^, Yongzhong Tong^1^, Huifang Chen^1^, Xuemei Sun^2^, Jing Yang^4^, Naoji Matsuhisa^5^, Huisheng Peng^2^*, Meifang Zhu^1^*, and Shaowu Pan^1^*

^1^State Key Laboratory for Modification of Chemical Fibers and Polymer Materials, College of Materials Science and Engineering, Donghua University, Shanghai 201620, China;

^2^State Key Laboratory of Molecular Engineering of Polymers, Department of Macromolecular Science, and Institute of Fiber Materials and Devices, Fudan University, Shanghai 200438, China;

^3^MOE Key Lab of Disaster Forecast and Control in Engineering, School of Mechanics and Construction Engineering, Jinan University, Guangzhou 510632, China;

^4^Department of Cardiology, Shanghai Xuhui Central Hospital, Zhongshan-Xuhui Hospital, Fudan University, Shanghai 200031, China;

^5^Research Center for Advanced Science and Technology, and Institute of Industrial Science, The University of Tokyo, 4-6-1 Komaba, Meguro-ku, Tokyo 153-8505, Japan

***Corresponding authors.** E-mail:

penghs@fudan.edu.cn; zhumf@dhu.edu.cn; pansw@dhu.edu.cn

**Experimental Section**

**Fabrication of CsAFS.** Stretchable fiber strain sensors were fabricated by depositing the active materials onto elastomer fibers. First, PDMS fibers were fabricated from the template method. Base and curing agent of PDMS prepolymer (Sylgard 184) were mixed at a weight ratio of 10:1, and the degassed PDMS prepolymer was transferred into the syringe and extruded into a Teflon tube (inner diameter: 0.8 mm) using an injection pump. Then, the PDMS prepolymer was cured at 100 °C for 3 hr to obtain PDMS fibers after demolding. In order to improve the surface activity of the PDMS fibers, they were treated by oxygen plasma (100 W, 30 s, 3 mbar). TPU fibers with a diameter of 0.9 mm were commercially available. The spraying ink is consisted of conductive materials and silane coupling agent. In detail, CNTs (length: 10 μm, diameter: 30-50 nm) and 3-aminopropyltriethoxysilane were added to anhydrous hexane at mass ratios from 1:2 to 1:8. The mixture was ultrasonically dispersed for 30 min and then quickly transferred to the spray gun (USTAR, S-130). Specifically, the nozzle of spray gun was perpendicular to the upper surface of the fiber. The ink was sprayed onto half of the fiber surface under a pressure of 1 MPa at a movement speed of 2 cm/s, and the distance between nozzle and the fiber was fixed at 5 cm. Due to the rapid volatilization of hexane, the CNTs and 3-aminopropyltriethoxysilane were successfully deposited onto the surfaces of elastic fibers, and a conductive sensing material layer was firmly anchored onto fiber surfaces after the hydrolysis and self-condensation of 3-aminopropyltriethoxysilane for 12 hr at room temperature, achieving CsAFS. The thickness of the sensing layer in fiber strain sensors was controlled by spraying times.

**Fabrication of CCFS.** CNTs/n-hexane solution with a concentration of 1.25 wt% CNTs was spraying onto PDMS fiber under a pressure of 1 MPa at a movement speed of 2 cm/s, and the distance between nozzle and the fiber was fixed at 5 cm. CCFS was achieved by fully evaporating hexane at room temperature.

**Fabrication of CFFS.** CFFS were fabricated by extrusion and heat curing processes. In detail, CNTs was added to the toluene-diluted PDMS base with a concentration of 3 wt%. The toluene was volatilized by continuous stirring under heat. Then, the curing agent was uniformly mixed with CNTs/PDMS base solution. The final mixture was transferred to a syringe with a needle of 20 G. CNTs/PDMS fibers were prepared by extruding the aforementioned mixture into a 200 ℃ oil bath for an instantaneous curing process. CFFS was obtained by removing surface oil using isopropyl alcohol.

**Characterization.** The structure was observed using cold field emission scanning electron microscope (SU-8010, Hitachi). FT-IR spectra were recorded using Fourier transform infrared spectrometer (Nicolet iS50, PerkinElmer). The fluorescence images were obtained by fluorescence microscope (BX51, Olympus). The mechanical properties of the samples were tested by universal tensile testing machine (UTM2103, Shenzhen Suns technology). The electrical resistance of fiber sensor was recorded by a source-meter (4200A-SCS, Keithley).

**Measurement of baPWV.** To measure the baPWV values of subjects, we recorded BPW and APW simultaneously using two CsAFS. The effective length of the fiber sensor is around 1 cm. A fiber sensor was attached to the brachial artery of the left arm, while another was attached to the ankle artery in left ankle using thin medical PU tapes. The time intervals (*∆T*) were obtained by calculating the time delay from the arrival of pulse wave at these two points, and the values of *L_a_* and *L_b_* can be obtained from the height of subjects. Finally, the baPWV values of subjects were calculated from following equation: $\text{baPWV}\text{ }\text{= (}\text{L}_{\text{a}}\text{-}\text{L}_{\text{b}}\text{)/}\text{∆T}$. As a contrast, the baPWV values of subjects were also measured by a medical Omron arteriosclerosis detector (BP-203RPE Ⅲ). An informed written consent from all participants was obtained prior to the research.

**Demonstration of Respiratory Monitoring System.** The stretchable fiber strain sensor with a length of 3 cm was integrated into the sweater, which was located at the abdomen position to monitor the respiratory state. The customized circuit board was used to connect the fiber strain sensor to collect, process, and wirelessly transfer the data to a smart phone for displaying health information.

**
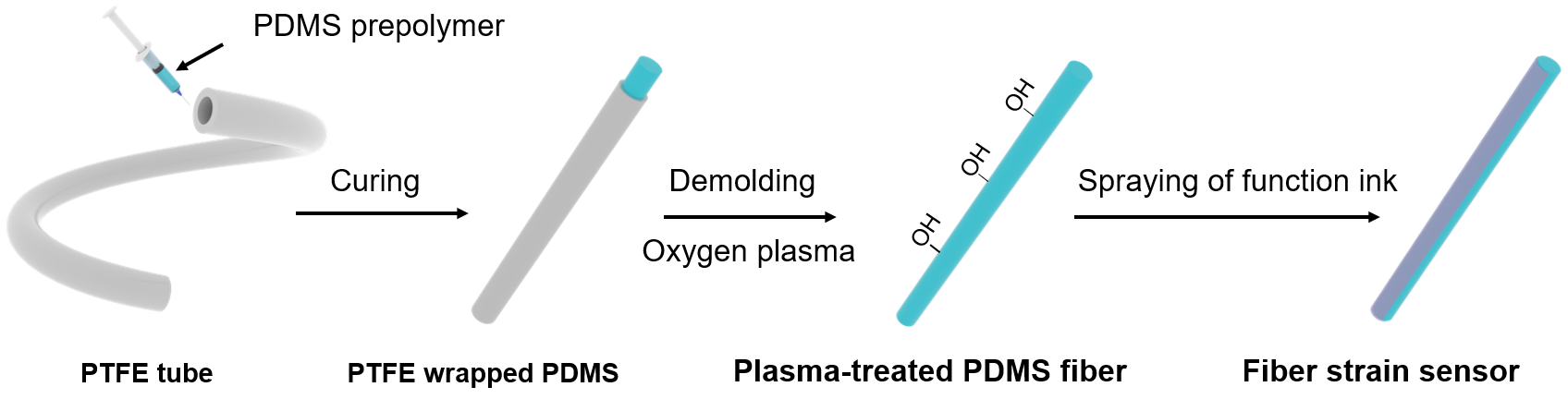
**

**Figure S1.** Schematic illustration of the fabrication process of stretchable fiber strain sensors.

**
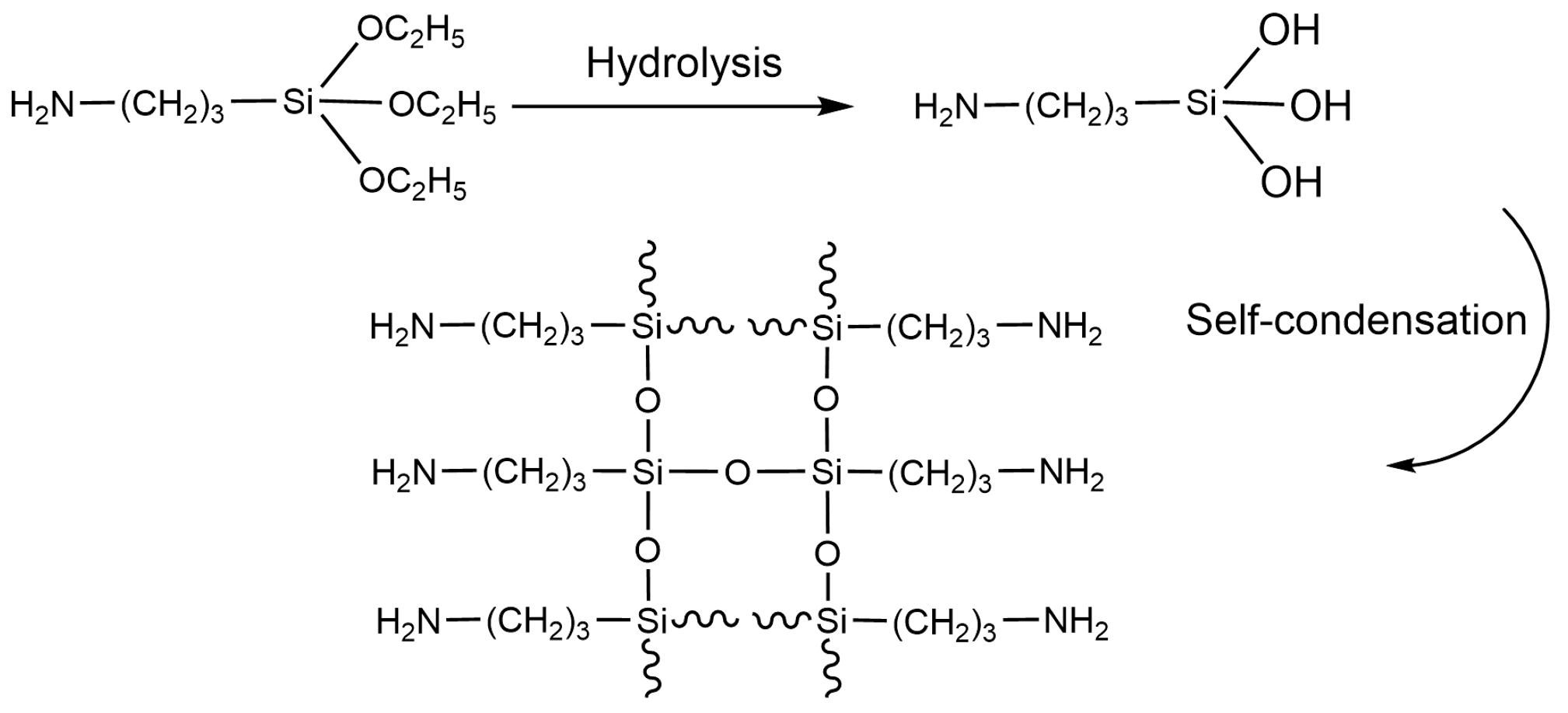
**

**Figure S2.** The hydrolysis and self-condensation process of 3-aminopropyltriethoxysilane.

**
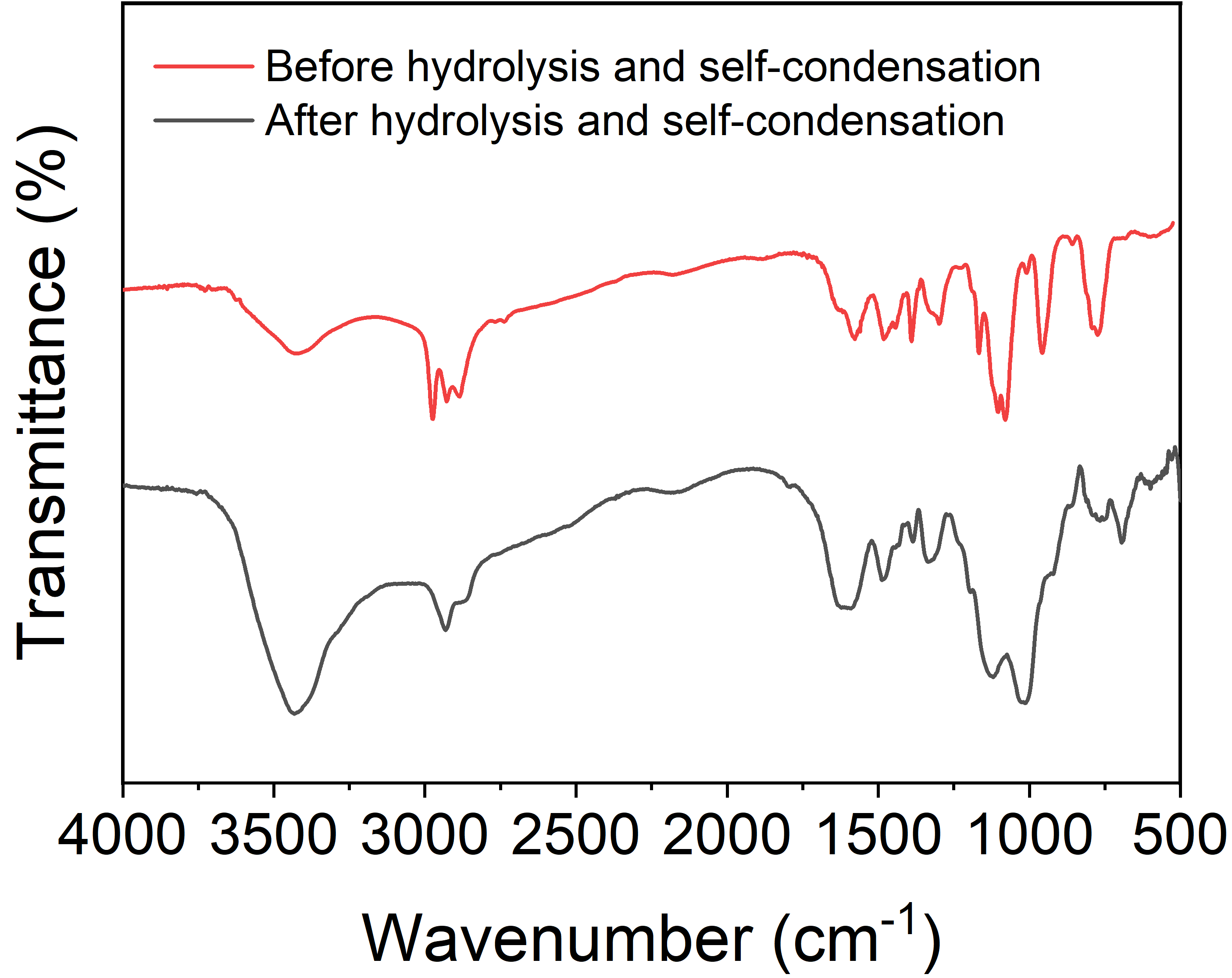
**

**Figure S3.** FTIR spectra of 3-aminopropyltriethoxysilane before and after hydrolysis and self-condensation.

**
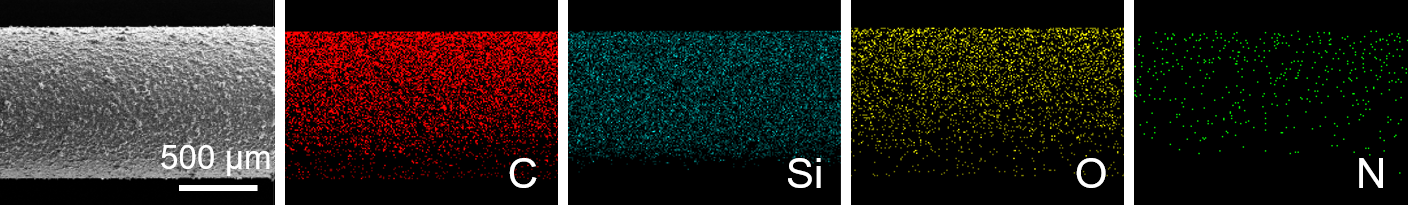
**

**Figure S4.** Element mapping images of CsAFS.

**
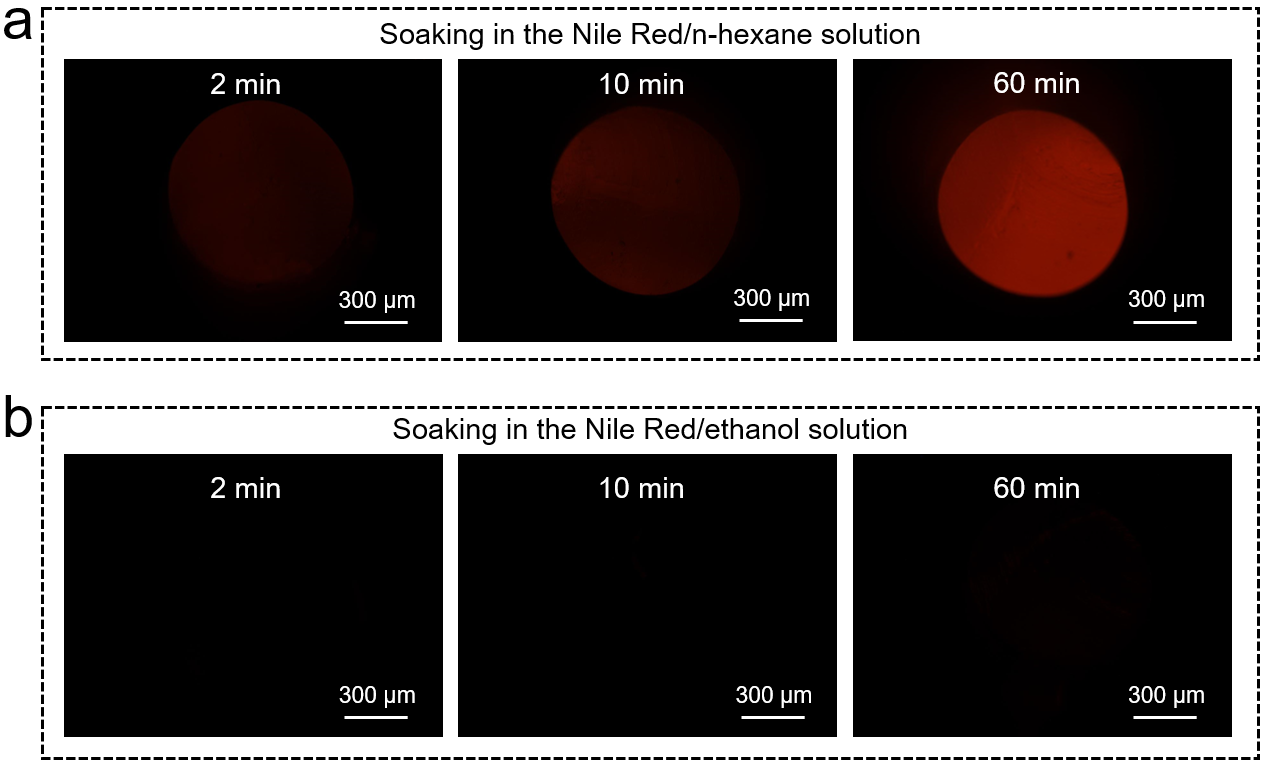
**

**Figure S5.** Fluorescence microscope images of PDMS fibers soaked in Nile Red/n-hexane solution (a) and Nile Red/ethanol (b) for different times. PDMS readily swells in n-hexane but is difficult to swell in an ethanol solution.

**
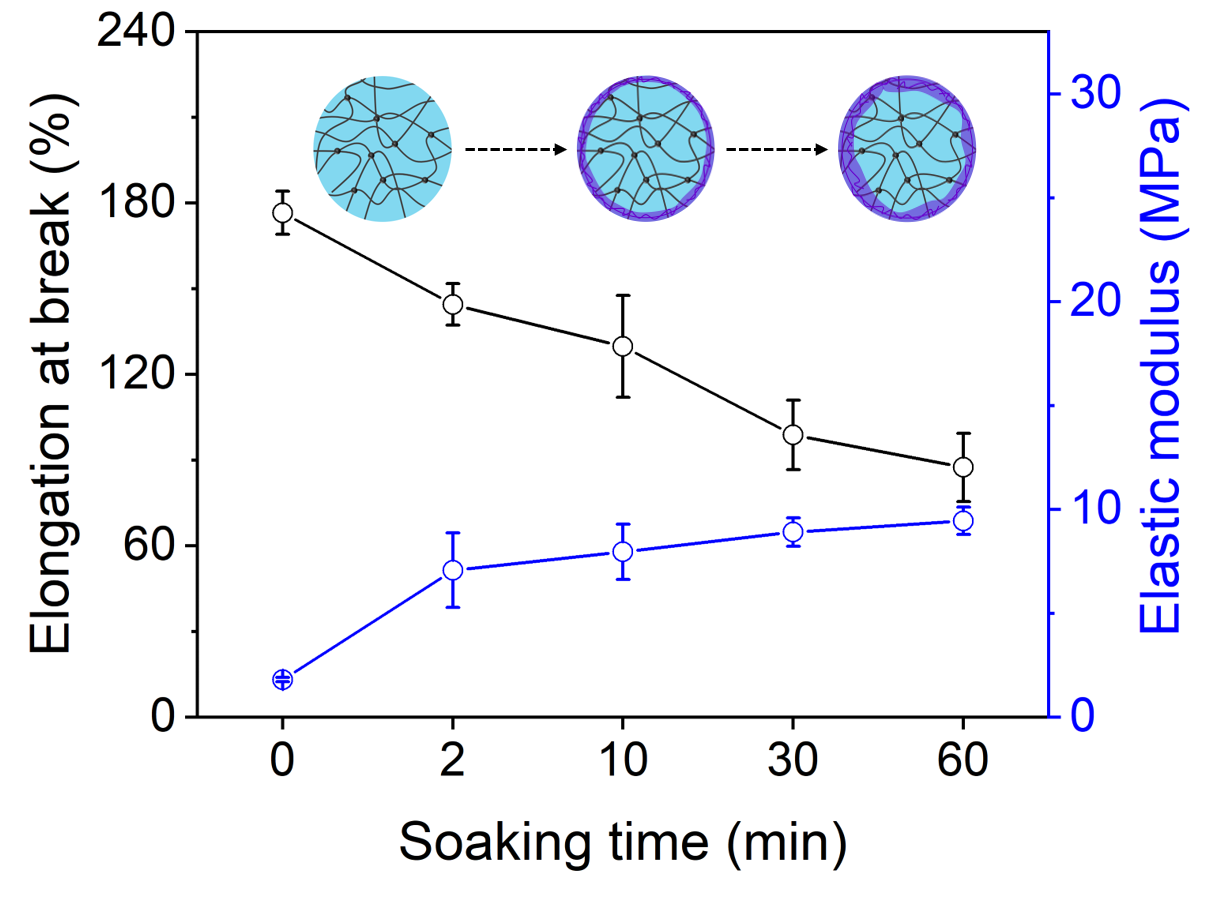
**

**Figure S6.** Mechanical properties of PDMS fibers soaked in 3-aminopropyltriethoxysilane/n-hexane solution for different times. The concentration of 3-aminopropyltriethoxysilane in n-hexane was 4.7 wt%. The modified PDMS fibers were washed with ethanol after the volatilization of n-hexane. Fibers were tested after the hydrolysis and condensation of 3-aminopropyltriethoxysilane. Inset demonstrates the gradual diffusion of 3-aminopropyltriethoxysilane into PDMS network as the soaking time increases.

**
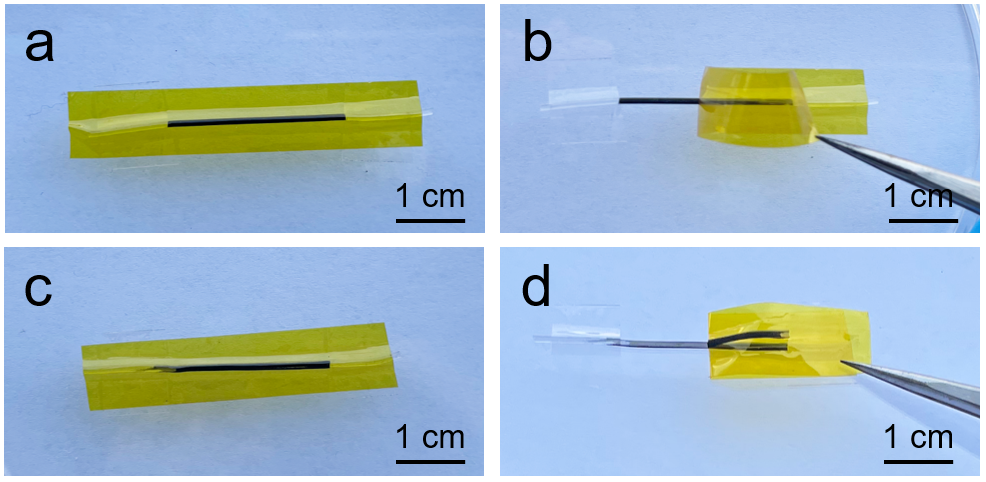
**

**Figure S7.** Optical images of adhesion strength comparison in CsAFS (a and b) and CCFS (c and d). The polyimide tape was firstly attached to fiber strain sensors (a and c) and then was peeled off (b and d) to qualitatively show the adhesion strength between the sensing layer and PDMS fiber. The mass ratio of CNTs to 3-aminopropyltriethoxysilane was 1:8 in CsAFS.


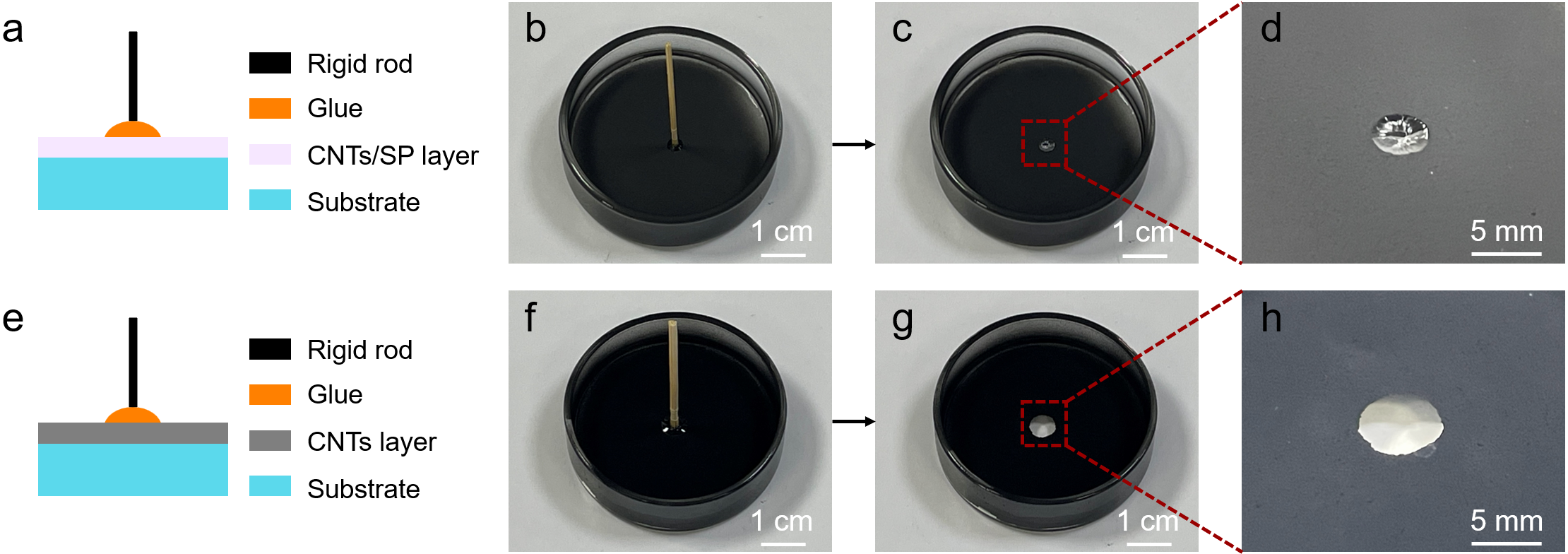


**Figure S8.** Schematic illustration of the tensile test using a rigid rod and epoxy resin as glue. a-d) The CNTs/SP-anchored PDMS film. e-h) The CNTs-coated PDMS film.


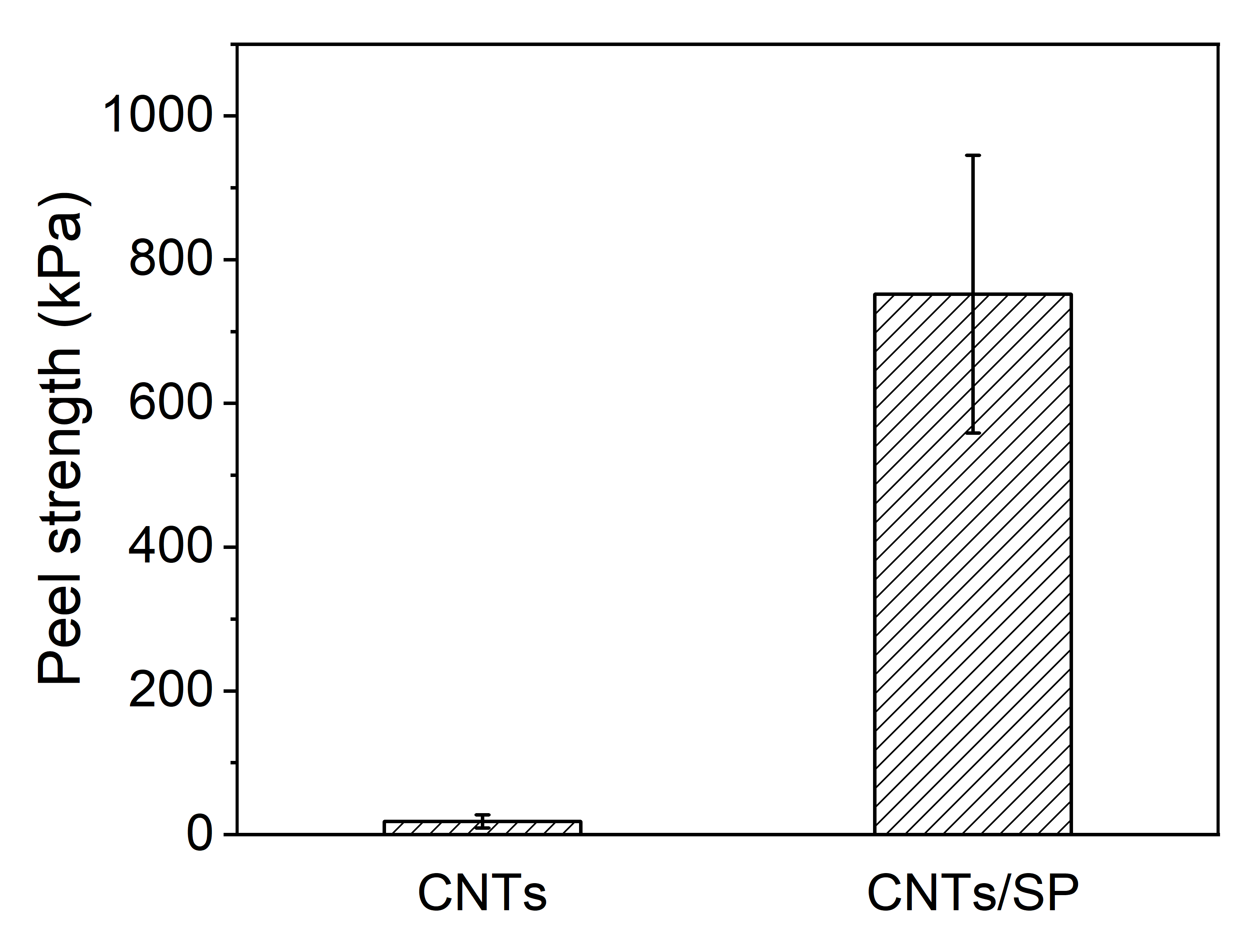


**Figure S9.** The peel strength in CNTs-coated PDMS film and CNTs/SP-anchored PDMS film.


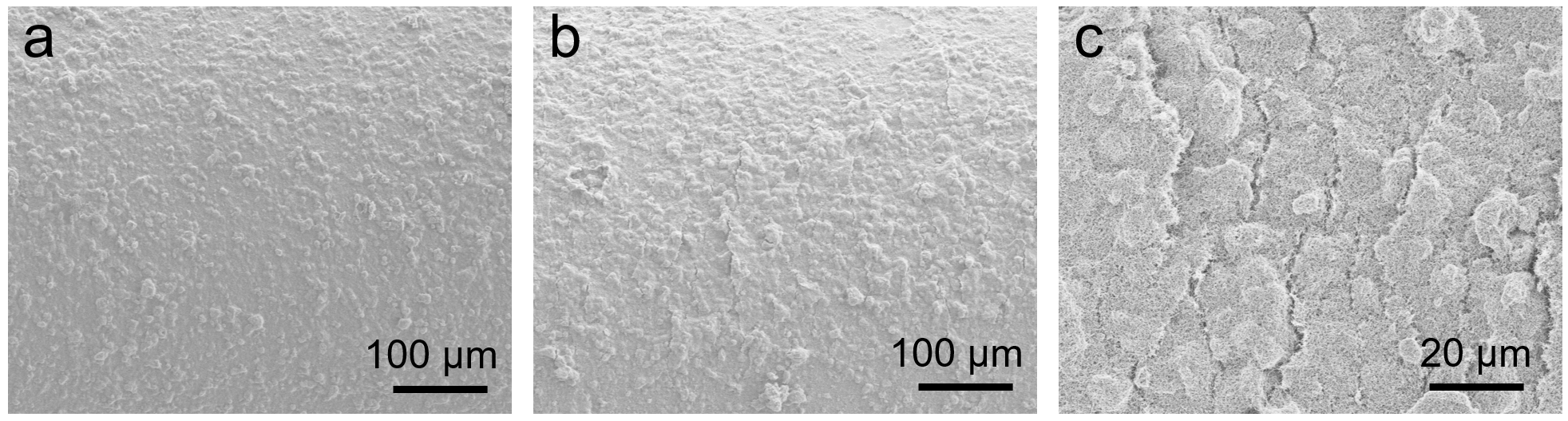


**Figure S10.** SEM images of CCFS under tensile strains of 0% (a) and 50% (b and c).


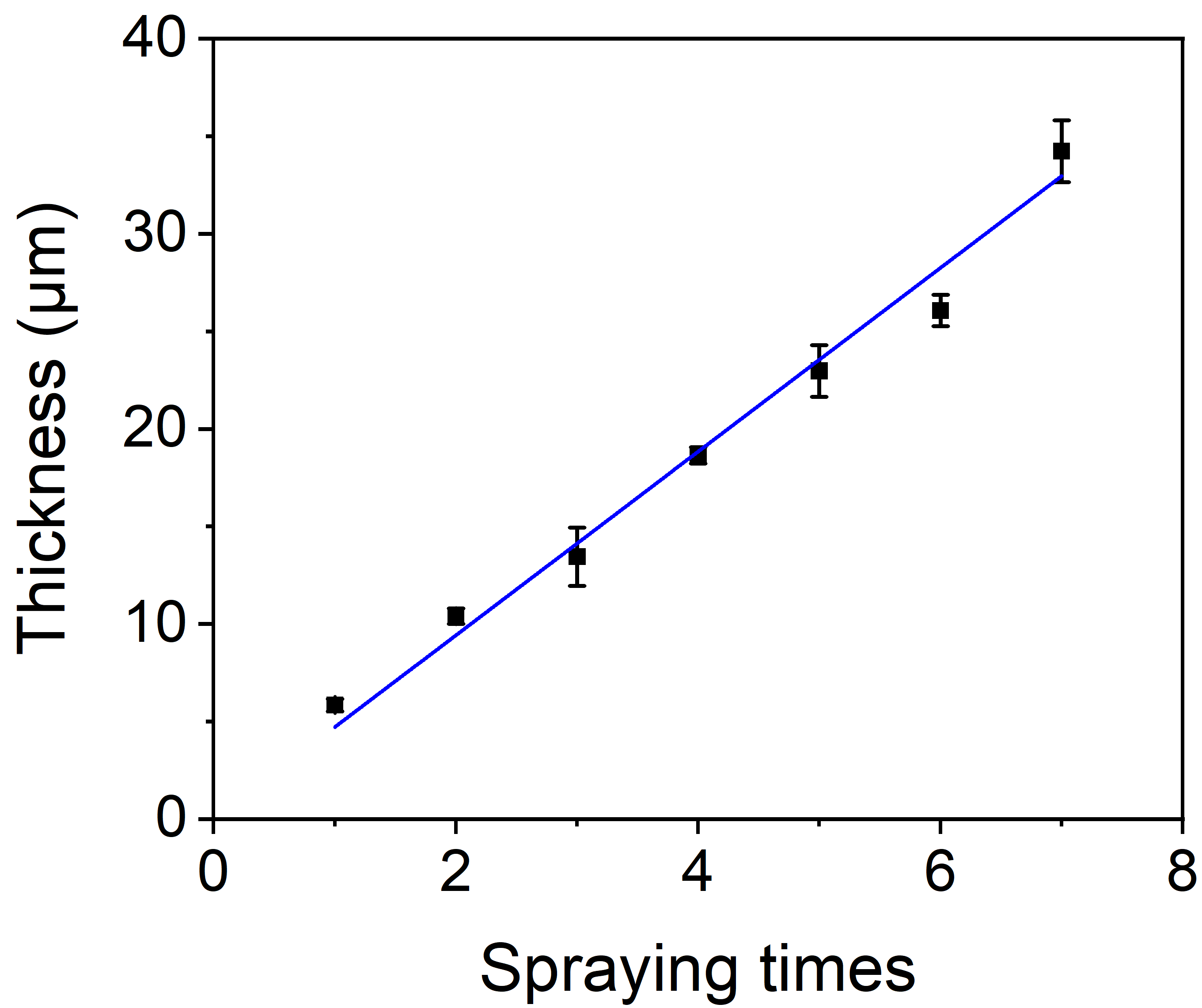


**Figure S11.** The thickness of CNTs/SP sensing layer with different spraying times. The blue line is a fitting curve, and adjusted *R^2^* is 0.994.


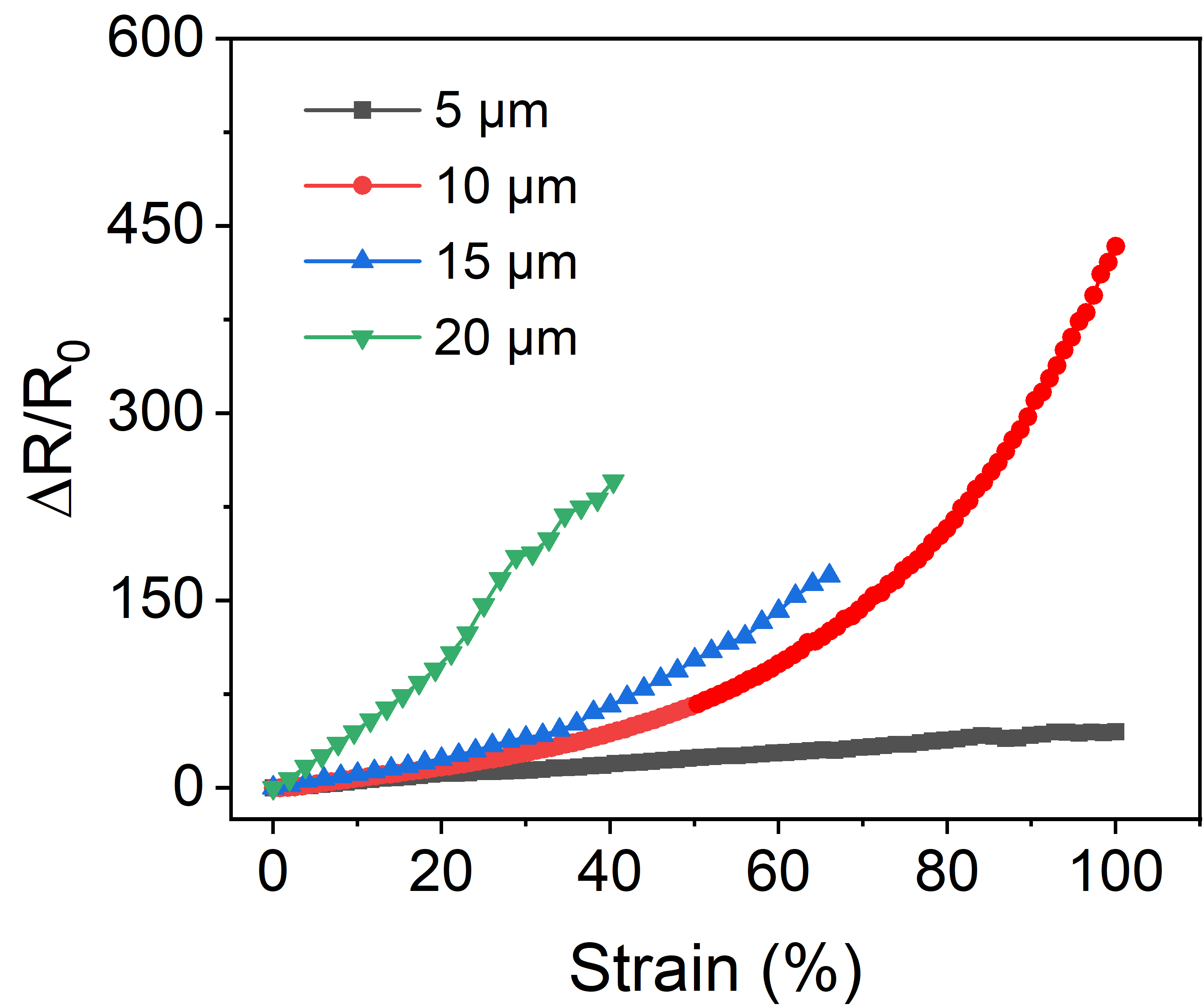


**Figure S12.** Relative resistance change curves of CsAFS with different thicknesses of sensing layer.


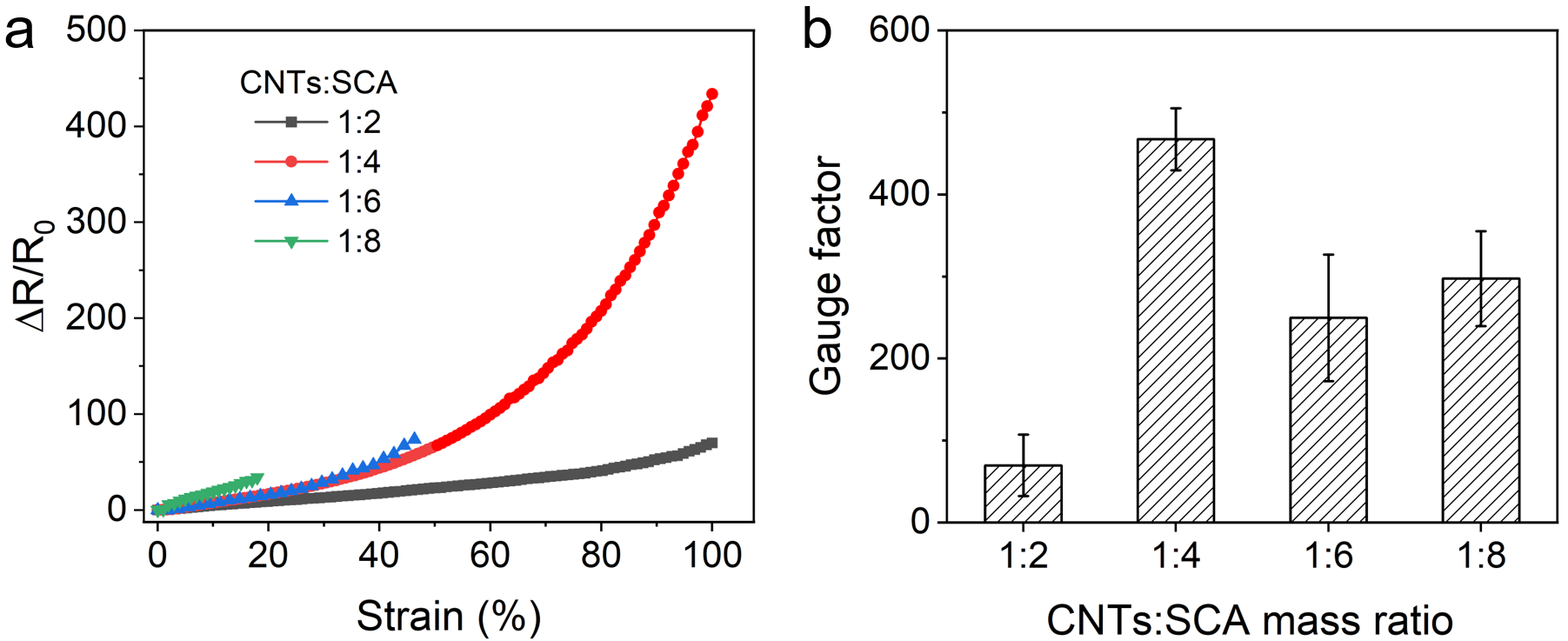


**Figure S13.** a) Relative resistance change curves of CsAFS with different mass ratios of CNTs and silane coupling agent (SCA) of 3-aminopropyltriethoxysilane. b) Sensitivity comparison of fiber sensors with different ratios of CNTs and SCA.

**
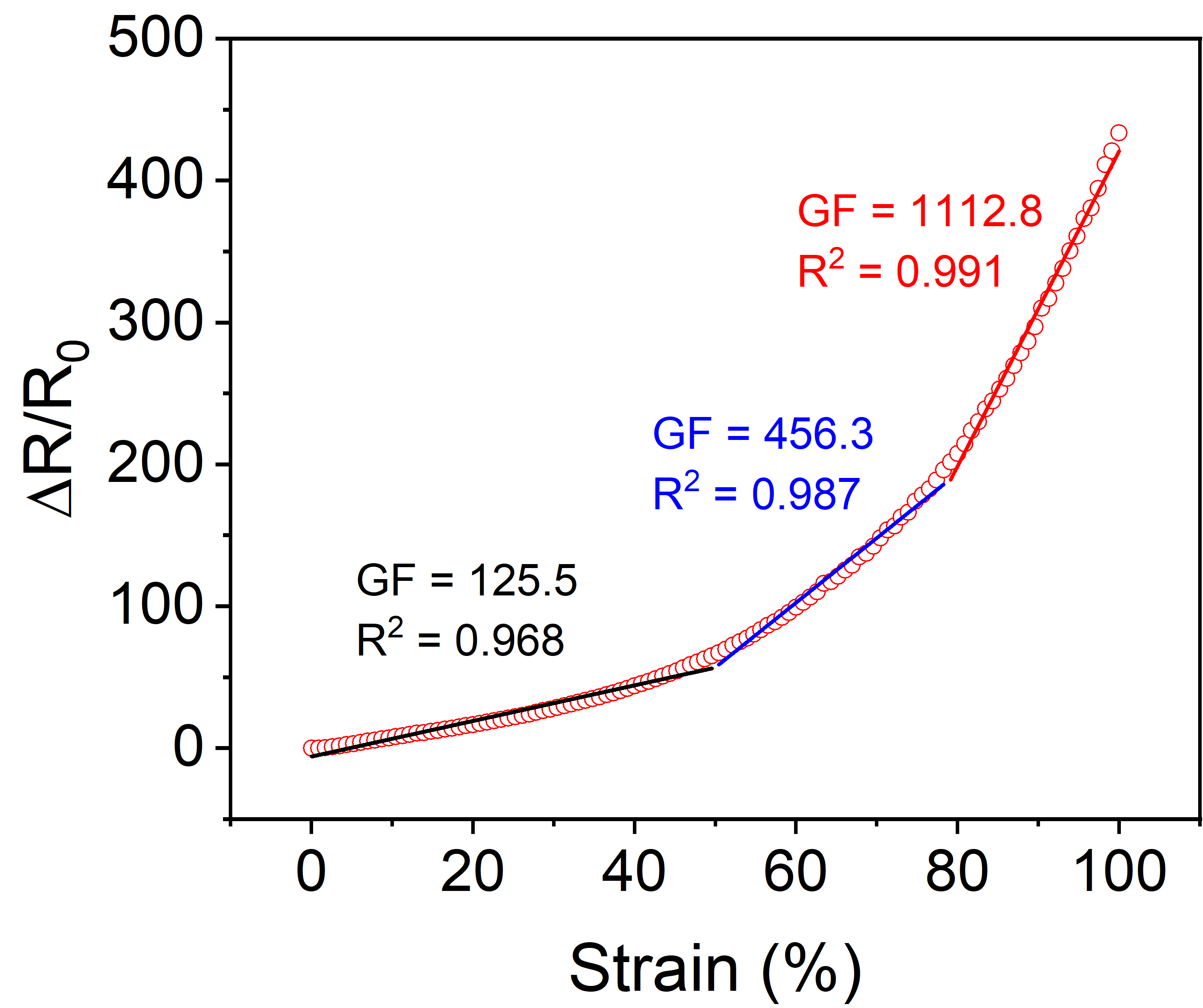
**

**Figure S14**. The sensitivity of CsAFS within the three linearity ranges.


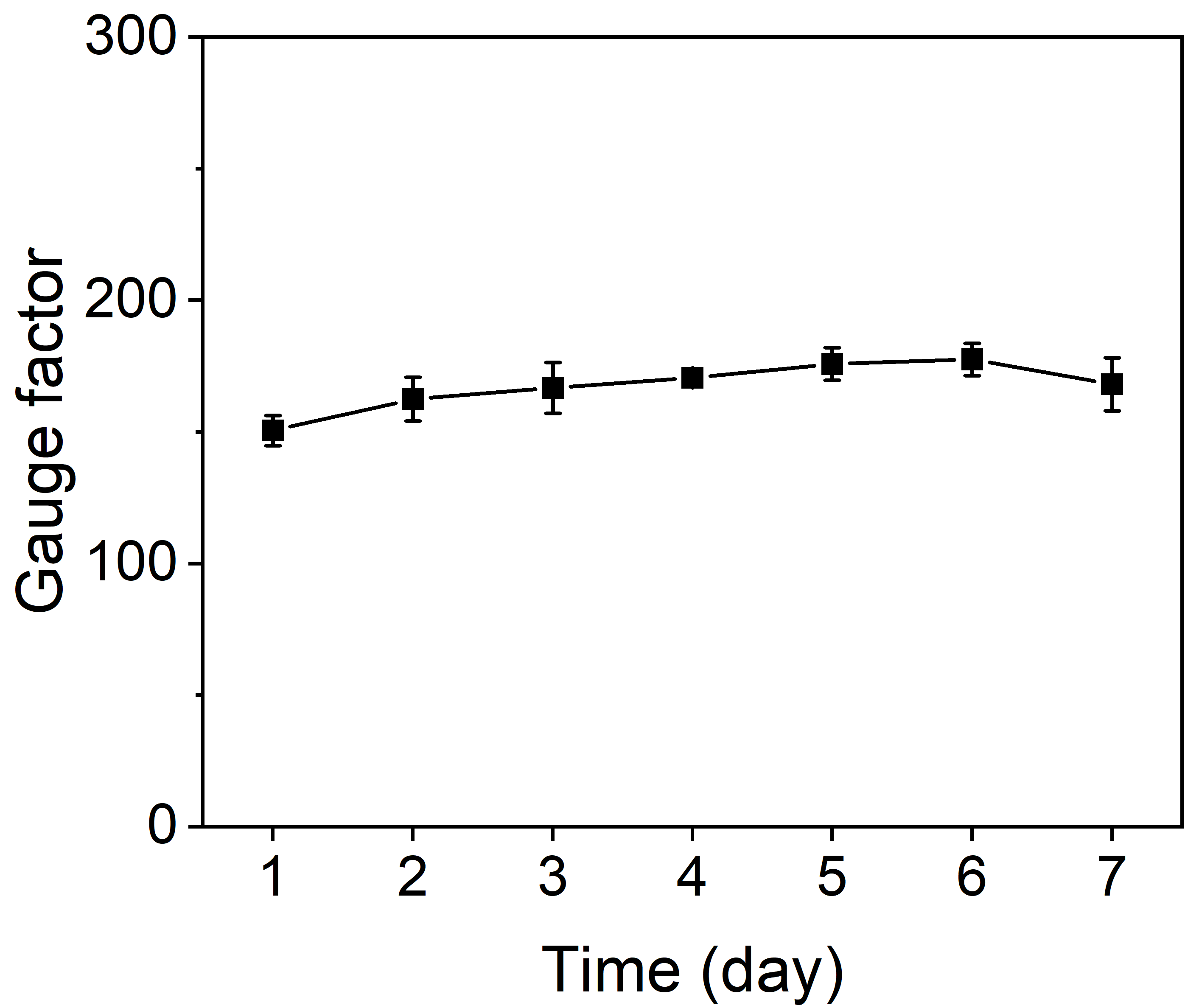


**Figure S15.** The sensitivity of CsAFS tested over one week under an applied strain of 50%.


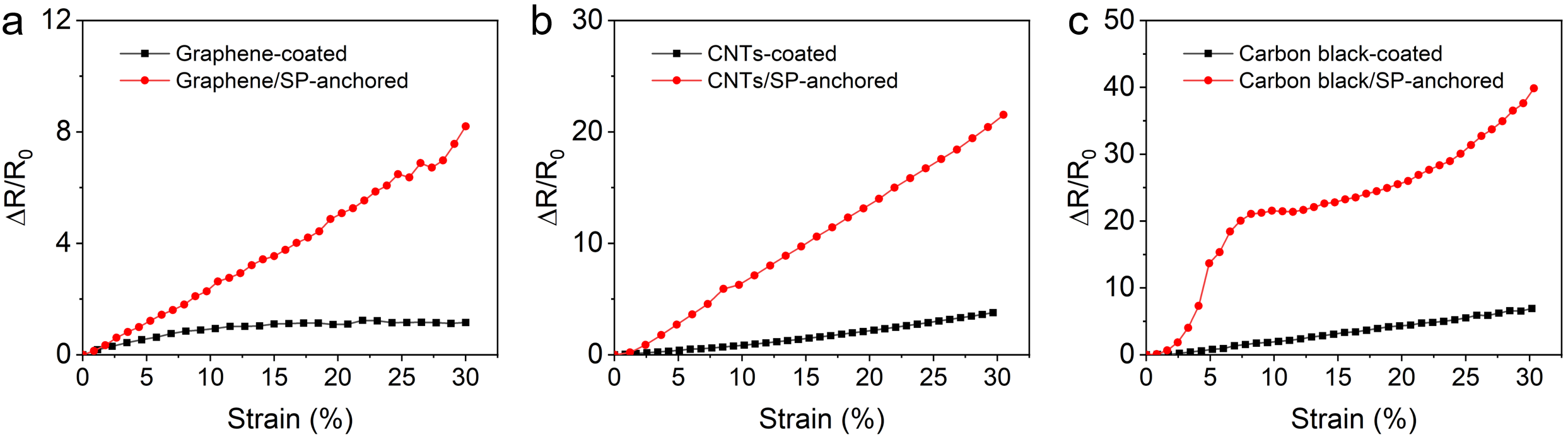


**Figure S16.** Relative resistance changes of fiber strain sensors based on SP anchoring sensing layer and surface coating using different conductive materials, including graphene (a), CNTs (b), and carbon black (c); the substrate is PDMS fiber.


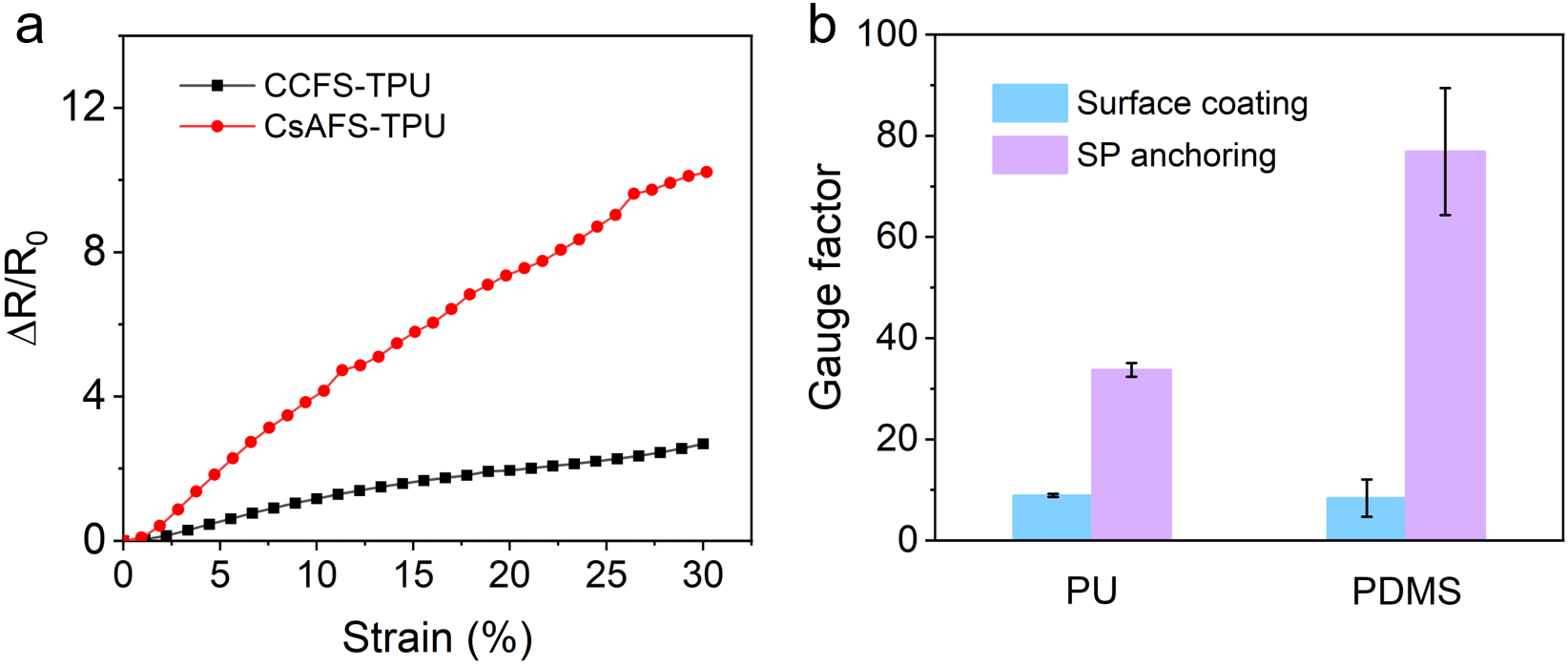


**Figure S17.** a) Relative resistance changes of fiber strain sensors based on SP anchoring layer and surface coating using TPU fiber substrate; the conductive material is CNTs. b) Sensitivity comparison of fiber sensors based on SP anchoring layer and surface coating using different fiber substrates; the conductive material is CNTs, and tensile strain is 30%.


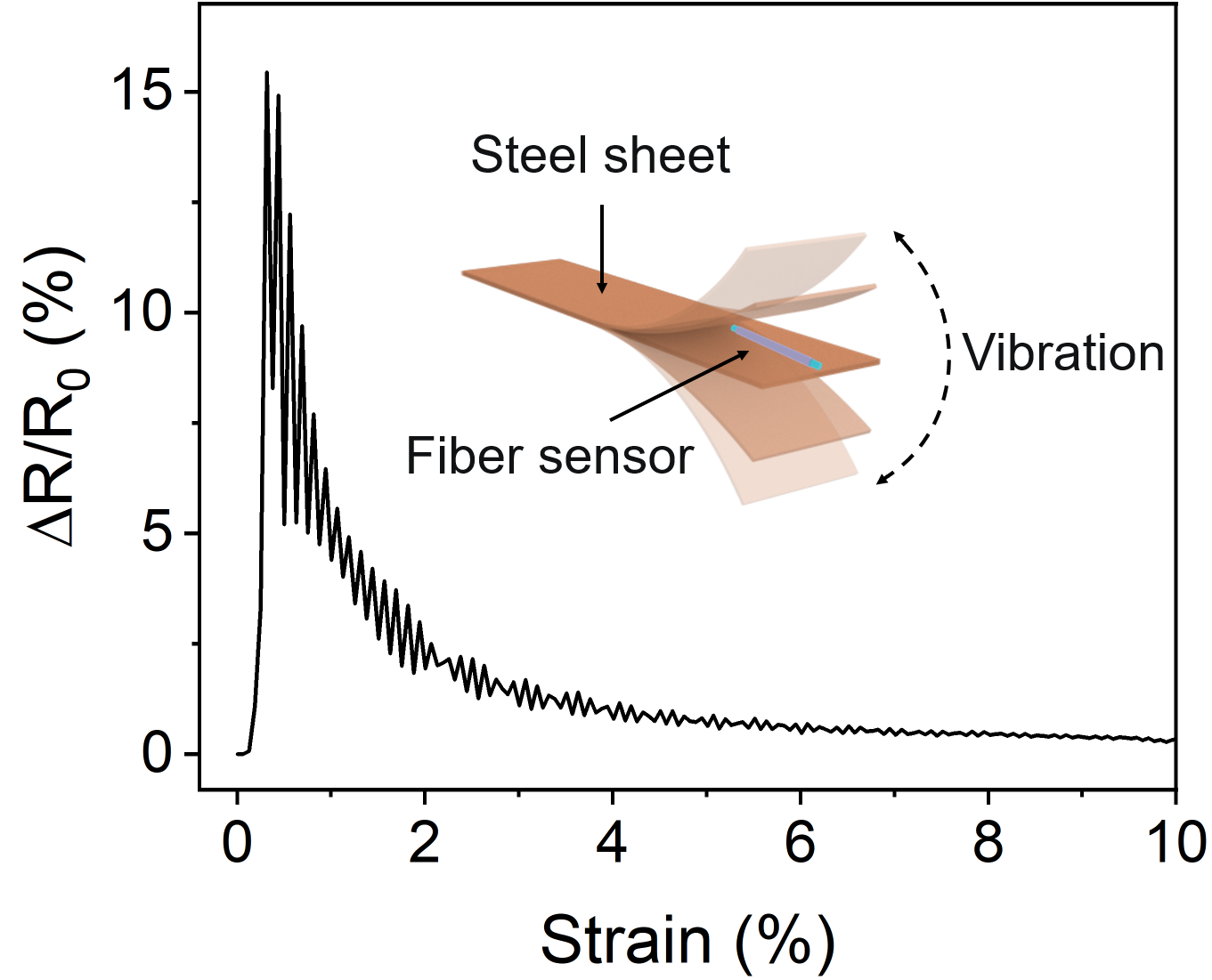


**Figure S18.** Damping vibration detected by CsAFS. Inset shows the testing schematic diagram.

**
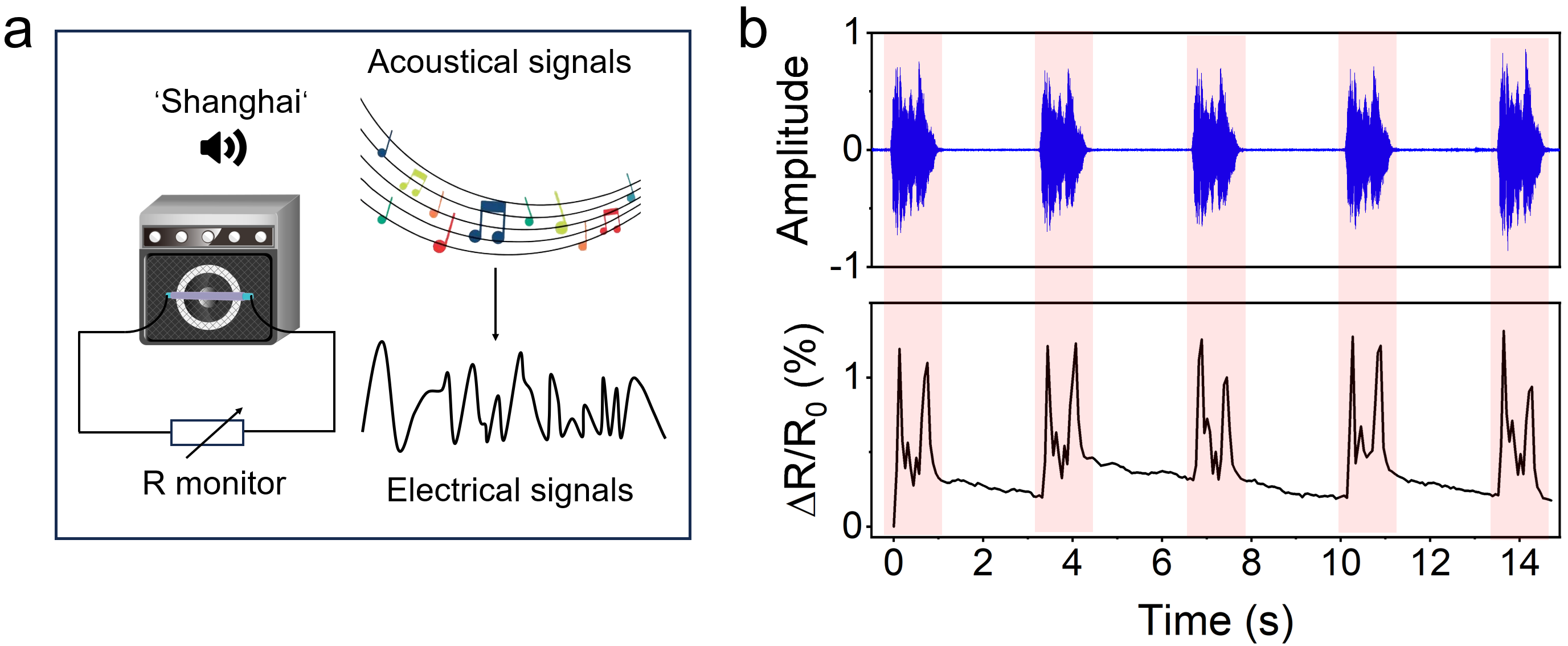
**

**Figure S19.** a) The schematic diagram for CsAFS converting audio signals from a loudspeaker into electrical signals. b) The audio signals of the sound system emitting the pronunciation of “Shanghai” and the corresponding electrical signals detected from CsAFS.


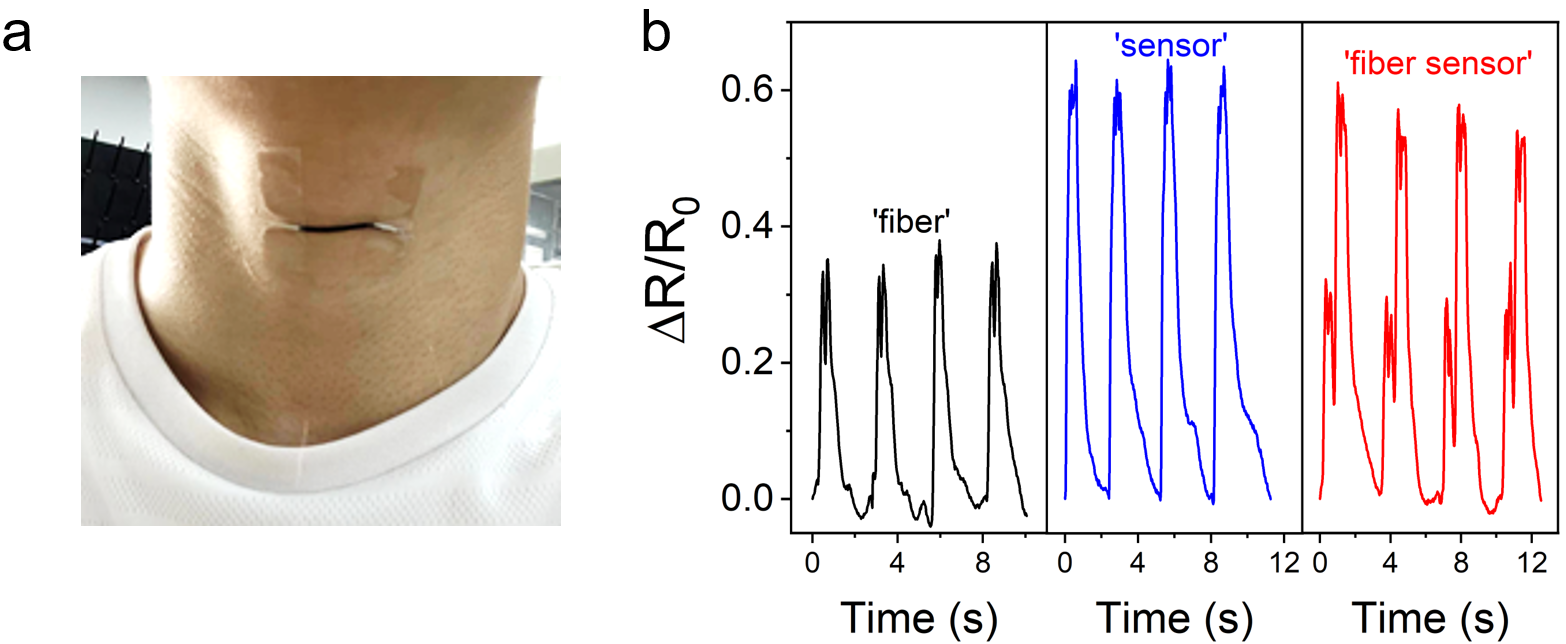


**Figure S20.** a) Photograph of CsAFS attached onto the throat of a volunteer. b) Signals detected by CsAFS when the volunteer spoke words of “fiber”, “sensor”, and “fiber sensor”.


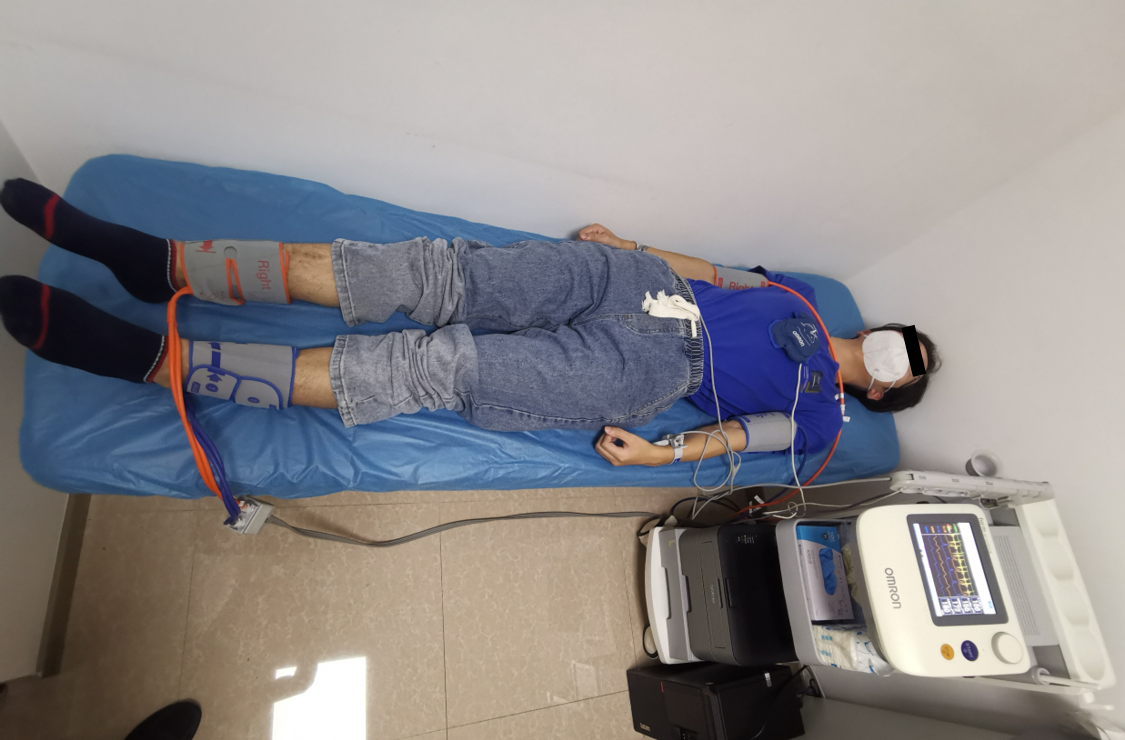


**Figure S21.** Photograph of the subject under baPWV measurement using the Omron arteriosclerosis detector (BP-203RPE Ⅲ).


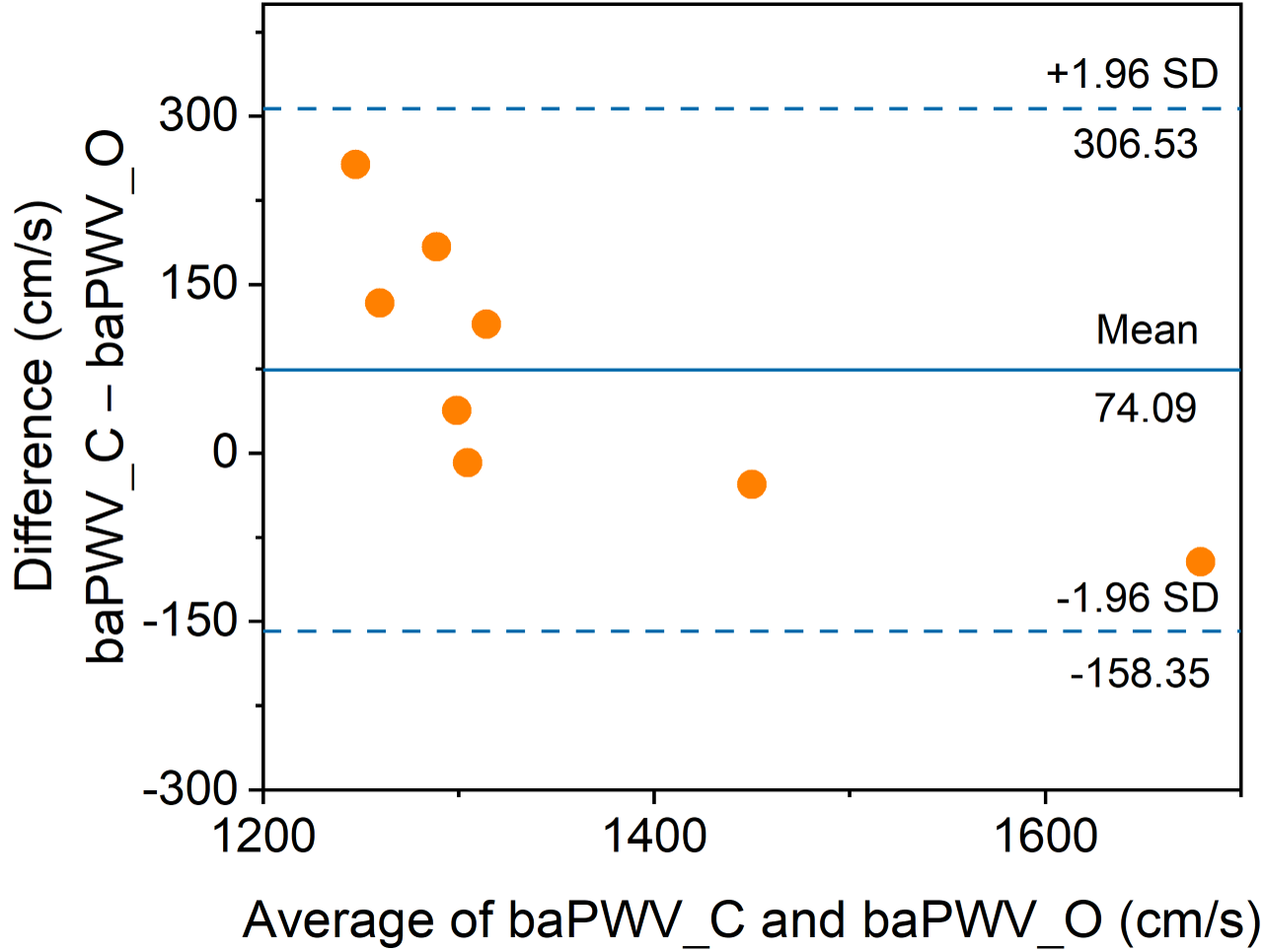


**Figure S22.** Bland-Altman plot of the differences between baPWV measured by CsAFS (baPWV_C) and baPWV measured by Omron arteriosclerosis detector (baPWV_O).


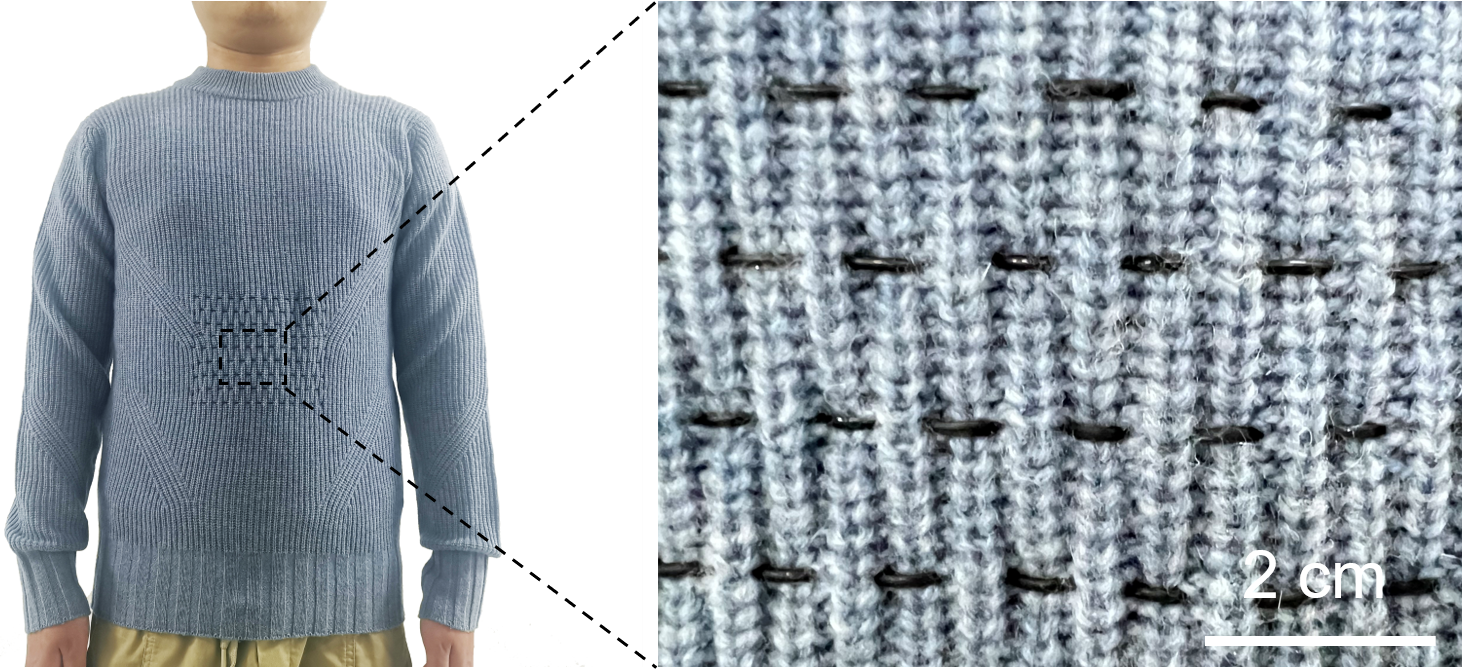


**Figure S23.** Photograph of CsAFS integrated into a sweater.


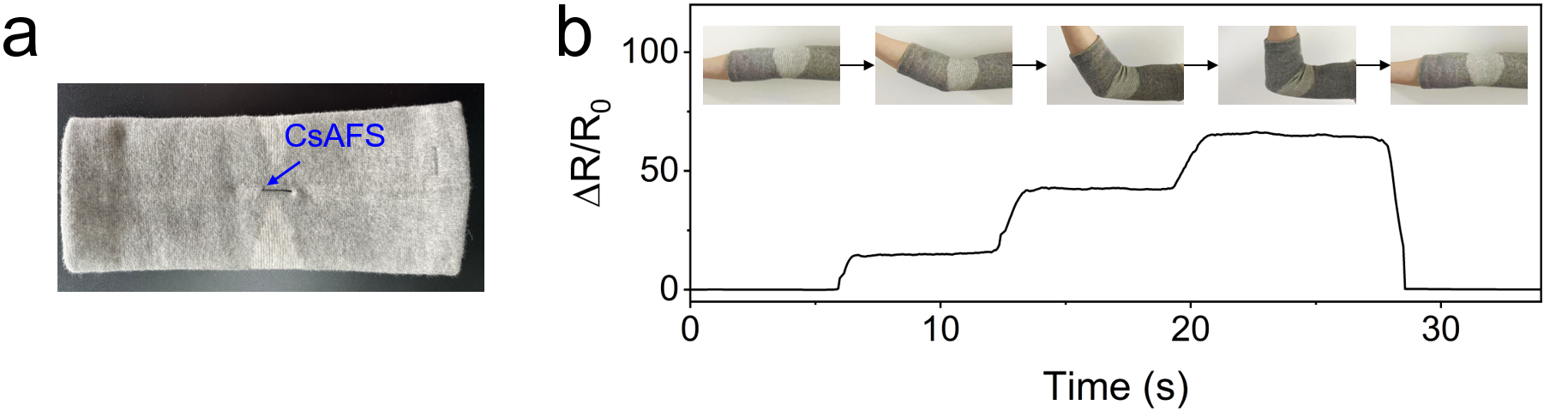


**Figure S24**. a) A sports sleeve integrated with a CsAFS. b) Relative resistance changes of the fiber strain sensor during elbow bending. Inset: photos of elbow bent at different angles.


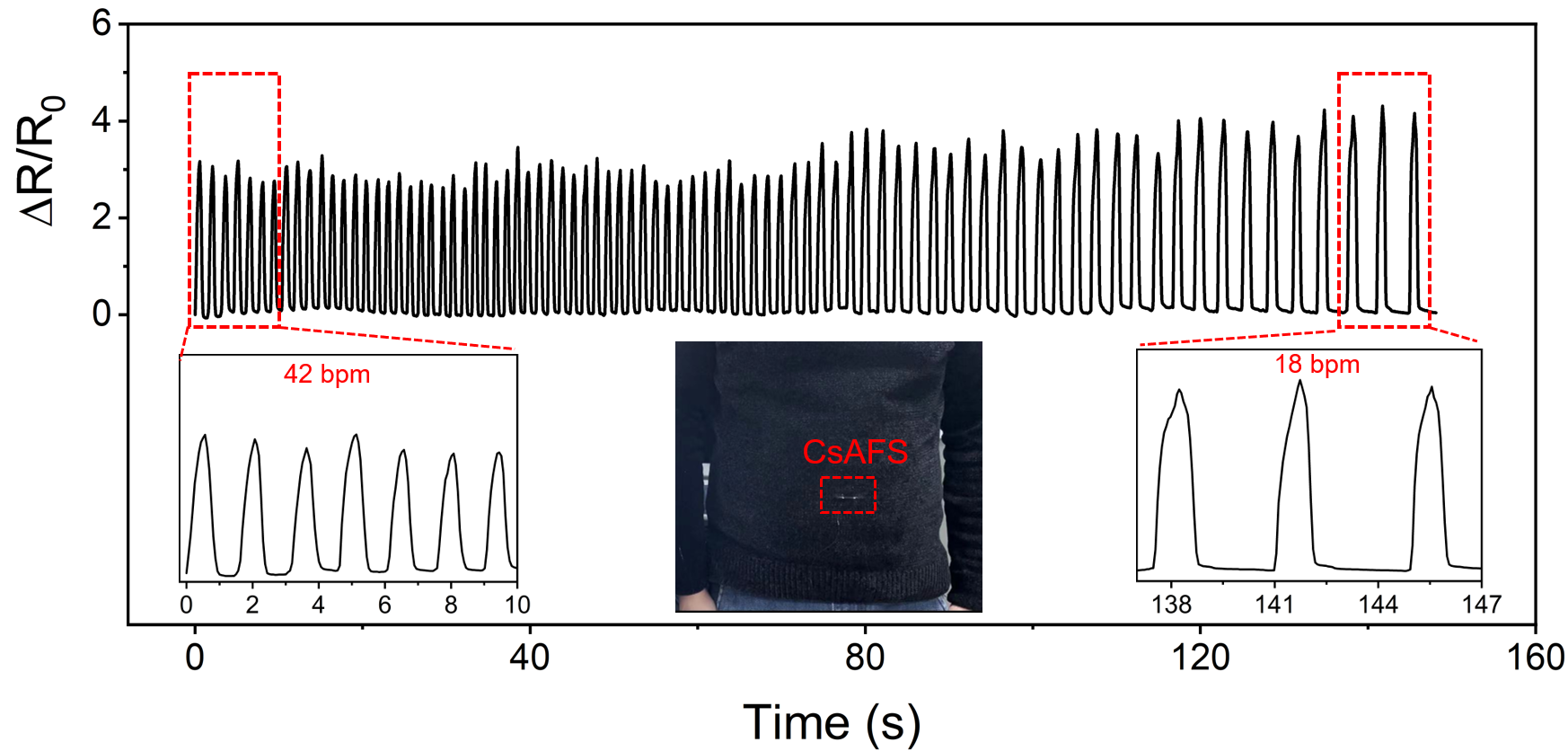


**Figure S25.** Relative resistance change curve of the volunteer's respiratory status recording after jogging of 20 minutes. Inset: photograph of CsAFS integrated into the sweater at the abdominal position.


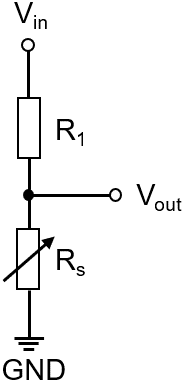


**Figure S26.** Circuit diagram of signal acquisition in the respiration monitoring system. R_1_ is a fixed resistor, Rs is the resistance of the fiber sensor.

**Table S1**. The detail performance comparison of fiber strain sensors in Figure 3b.

| Ref. | Gauge factor | Maximum Uniaxial strain |
| --- | --- | --- |
| S1 | 42.3 | 120% |
| S2 | 87.0 | 8% |
| S3 | 32.0 | 400% |
| S4 | 6.2 | 300% |
| S5 | 86.9 | 50% |
| S6 | 15.2 | 150% |
| S7 | 63.0 | 65% |
| S8 | 3.0 | 100% |
| S9 | 37.3 | 30% |
| This work | 433.6 | 100% |

**Supplementary References**

1. Liu Z, Qi D, Hu G *et al.* Surface strain redistribution on structured microfibers to enhance sensitivity of fiber-shaped stretchable strain sensors. *Adv Mater* 2018; **30**: 1704229.
2. Huang T, He P, Wang R *et al.* Porous fibers composed of polymer nanoball decorated graphene for wearable and highly sensitive strain sensors. *Adv Funct Mater* 2019; **29**: 1903732.
3. Gao J, Fan Y, Zhang Q *et al.* Ultra-robust and extensible fibrous mechanical sensors for wearable smart healthcare. *Adv Mater* 2022; **34**: 2107511.
4. Chen Z, Liu H, Lin X *et al.* Competitive proton-trapping strategy enhanced anti-freezing organohydrogel fibers for high-strain-sensitivity wearable sensors. *Mater Horizons* 2023; **10**: 3569-81.
5. Li X, Hua T, Xu B. Electromechanical properties of a yarn strain sensor with graphene-sheath/polyurethane-core. *Carbon* 2017; **118**: 686-98.
6. Liao X, Liao Q, Zhang Z *et al.* A highly stretchable ZnO@fiber-based multifunctional nanosensor for strain/temperature/uv detection. *Adv Funct Mater* 2016; **26**: 3074-81.
7. Zhai H, Xu L, Liu Z *et al.* Twisted graphene fibre based breathable, wettable and washable anti-jamming strain sensor for underwater motion sensing. *Chem Eng J* 2022; **439**: 135502.
8. Ge J, Sun L, Zhang F-R *et al.* A stretchable electronic fabric artificial skin with pressure-, lateral strain-, and flexion-sensitive properties. *Adv Mater* 2016; **28**: 722-8.
9. Yan T, Zhou H, Niu H *et al.* Highly sensitive detection of subtle movement using a flexible strain sensor from helically wrapped carbon yarns. *J Mater Chem C* 2019; **7**: 10049-58.
